# Making community-based health planning and services work: Staffing, accountability and digital integration for quality primary health care in Northern Ghana

**DOI:** 10.1371/journal.pone.0341176

**Published:** 2026-02-02

**Authors:** Dennis Chirawurah, Felix Achana, Abdou Orou-Seko, Joyce Aputere Ndago, Colette Santah, Vida Nyagre Yakong, Stephen Apanga, Martin Nyaaba Adokiya

**Affiliations:** 1 Department of Environmental and occupational health, School of Public Health, University for Development Studies, Tamale, Ghana; 2 Department of Epidemiology, Biostatistics and Disease Control, School of Public Health, University for Development Studies, Tamale, Ghana; 3 Department of Epidemiology and Biostatistics, School of Public Health, University of Technology and Applied Sciences, Navrongo, Ghana; 4 Research Laboratory in Aquaculture and Aquatic Ecotoxicology, University of Parakou, Parakou, Benin; 5 Department of Social and Behavioral Change, School of Public Health, University for Development Studies, Tamale, Ghana; 6 Department of Social and Behavioral Sciences, School of Public Health, University of Ghana, Legon; 7 Department of Preventive Health Nursing, School of Nursing and Midwifery, University for Development Studies, Tamale, Ghana; 8 Department of Community Health and Preventive Medicine, School of Medicine, University for Development Studies, Tamale, Ghana; Kintampo Health Research Centre, GHANA

## Abstract

**Background:**

Strengthening primary health care through Ghana’s Community-based Health Planning and Services (CHPS) strategy depends on functional community structures, responsiveness, and integration into health information systems. However, the extent to which CHPS zones use the Ghana Community Scorecard (CSC) to promote accountability, equity, and service improvement remains unclear. We assessed CHPS staff categories and functionality in Northern region of Ghana and their ability to support Community Health Management Committees (CHMCs) in health facility assessments, Community Health Action Plans, and updating of results into existing digital platforms.

**Method:**

A cross-sectional design combined quantitative surveys and qualitative interviews across 86 CHPS zones in six districts between March 13–20, 2024. Analysis focused on staff categories, functionality, CSC trainings, facility assessment, utilization of results, feedback mechanisms, and service improvements. Qualitative data explored barriers and enablers shaping CHPS performance.

**Results:**

The 86 CHPS zones employed 549 health workers, predominantly female (51%). Categories included Community Health Officers (5%), midwives (17%), registered nurses (14%), enrolled nurses (33%), community health nurses (27%) and others (5%). Overall, 96% of CHPS zones were functional based on staff, CHMCs, volunteers, equipment, and service provision. About 88% had basic equipment. Services include outreach, home visits, minor illness treatment, antenatal care, and referrals. CSC training reached 41% of Community Health Officers and other health workers. Only 36% of the CHPS zones uploaded facility assessment results to existing digital platforms, and 46.5% implemented improvements from CSC recommendations. High-performing districts benefitted from adequate staffing, training, Non-Governmental Organization support, and community mobilization. Barriers included limited training coverage, exclusion of midwives and nurses from training, and persistent Gender, Equity and Social Inclusion (GESI) gaps.

**Conclusion:**

CHPS zones show functionality but face challenges in staff capacity, training, and digital integration. Gaps in inclusivity and equipment provision limit effectiveness. Scaling-up training, strengthening human resources, improving basic equipment provision, and embedding GESI are essential to ensure CHPS zones deliver equitable, accountable, and quality services.

## Introduction

Community-oriented primary health care (PHC) is central to achieving Universal Health Coverage (UHC) and the health-related Sustainable Development Goals (SDGs) [1]. Community health worker (CHW) programs and decentralized delivery models extend services to underserved populations, reducing maternal and child mortality and improving access to care, particularly in low- and middle-income countries (LMICs) [2,3]. Yet health care systems in many of these countries continue to face workforce shortages, inadequate deployment, weak accountability, limited gender and social inclusion, and inadequate digital integration; constraints that hinder PHC’s transformative potential [4,5].

Social accountability tools such as the Community Scorecard (CSC) can empower communities to assess services, strengthen provider responsiveness and trust, and improve utilization and outcomes when integrated within supportive governance and information systems [6]. Indeed, the CSC uses structured scoring, community provider interface meetings, and joint action planning to evaluate service quality and responsiveness. These steps create iterative feedback loops that strengthen transparency and provider accountability. When integrated into supportive governance structures and connected to functional information systems, the CSC can reinforce PHC delivery and enhance service utilization and health outcomes. Evidence from Uganda, Malawi, and Tanzania indicate that when adequately resourced and community-led, CSC processes can improve provider accountability, strengthen trust between communities and providers, and lead to measurable improvements in service utilization and health outcomes [7,8]. Yet, the sustainability and effectiveness of these vital mechanisms depend on their alignment with broader health system structures, supportive governance, digital data infrastructures, and inclusive approaches that prioritize gender, equity and social justice [8,9].

Ghana’s Community-based Health Planning and Services (CHPS) initiative is a well-established model of decentralized PHC. Community Health Officers (CHOs) and volunteers provide frontline services within designated zones [10,11]. As of 2020, a total of 2,523 trained CHOs were deployed across 5,062 functional CHPS zones with active community health committees, supported by 19,411 community health volunteers [12]. Empirical work has shown gains in antenatal care, skilled birth attendance, immunization, and other indicators [13,14]. Nonetheless, CHPS functionality varies by region. Persistent challenges include uneven distribution of skilled personnel, limited capacity for community participation, weak institutionalization of accountability tools, and gaps in digital health data use [4,15]. Gender, Equity and Social Inclusion (GESI) considerations are particularly salient: women’s access improves with gender-sensitive provision [16], while exclusion of youth, persons with disabilities, and minority ethnic groups from governance can depress service uptake [17]. Despite policy commitments to inclusion, operationalization remains uneven, with little empirical documentation of GESI-responsive practices within CHPS implementation. The expansion of digital health reporting platforms (DHIMS2 and RMNCAH) offers opportunities for better planning and monitoring, but newer community instruments such as the CSC and resulting Community Health Action Plans (CHAPs) are seldom integrated, reducing visibility and use for decision making [18]. Findings from Tanzania, Nigeria, and Zambia indicate that weak digital capacity and reliance on paper-based processes often limit the reach and effectiveness of CSC interventions [19,20].

Despite demonstrated value of CHPS in expanding PHC in Ghana, a several studies on CHPS, major knowledge gaps remain [14,15,21] regarding how frontline staffing patterns, community accountability tools interact to shape service functionality and digital reporting performance at CHPS level. Specifically, there is limited empirical evidence regarding (i) CHPS zone staffing and functionality in relation to community accountability tools, (ii) the development of GESI-responsive CHAPs, and (iii) the integration of CSC/CHAP outputs into digital health platforms to support responsive planning, decision-making and continuous quality improvement. Existing literature highlighted persistent deficits in equitable staff deployment, limited institutionalization of social accountability mechanisms, and weak integration of community-generated data into national digital platforms, However, very few prior studies have examined these dimensions simultaneously within the same operational context. This study therefore provides an integrated assessment of these gaps by assessing staff categories and CHPS functionality in six districts of Northern region of Ghana. It examines the capacity of CHOs and other health staff to use Ghana CSC, develop GESI-responsive CHAPs, and disseminate outputs through digital health reporting platform DHIMS2 and RMNCAH.

## Materials and methods

### Study setting

This study was conducted in three districts (Karaga, Mion, and Nanton) and three municipalities within the Northern Region (Yendi, Gushegu, and Sagnarigu). The purposive selection of districts and CHPS zones was guided by two considerations. First, within each, three sub-districts were selected, aligning with the zones of influence (predefined geographic areas where technical support to strengthen PHC and CHPS implementation are provided) under the USAID Quality Services for Health (Q4H) Activity. This was done to reflect varied CHPS performance contexts (high-performing, average, and low-performing settings). Second, within each district, CHPS zones were included only if they met core eligibility criteria: (i) presence of resident CHOs, (ii) active community health management structures, and (iii) ongoing implementation of core CHPS service components. In Ghana’s local government structure, districts and municipalities perform similar statutory functions but differ in demographic and service-delivery contexts. Districts are predominantly rural, with dispersed settlements, stronger community structures, and wider geographic catchment areas. PHC governance in these settings relies more on CHPS zone expansion, community health committees, and volunteer-led service delivery. In contrast, municipalities are more urbanized, characterized by higher population density, more diverse populations, and weaker traditional governance structures. These conditions make CHPS implementation more complex, reduce reliance on volunteerism, and require greater integration with facility-based services and digital reporting systems. CHPS zones represent geographic catchment areas served by resident CHOs, who provide services from local compounds and are supported by community health management structures [22]. With more than 210 CHPS and 5,000 community-based agents active, Northern region of Ghana has recorded notable improvements in health knowledge and care-seeking practices, particularly in relation to childhood illness management and disease awareness [23,24]. The Northern Region’s CHPS programme serves a predominantly rural population of approximately 2,275,197 people, with roughly 53.2% of residents living in rural areas [25]. Nevertheless, significant challenges to CHPS functionality persist. For example, in Yendi Municipality, implementation has often been fragmented, characterized by weak coordination among stakeholders and unclear focus, which negatively affects equity and maternal health outcomes [26]. Similarly, findings from Jirapa Municipality highlight critical gaps, including inadequate logistics, limited training and supervision, weak planning, low staff motivation, and poor community engagement, all of which constrain effective delivery of maternal and child nutrition service [27].

### Overview of USAID Quality Services of Health Activity (Q4H) baseline assessment

**Fig 1:**
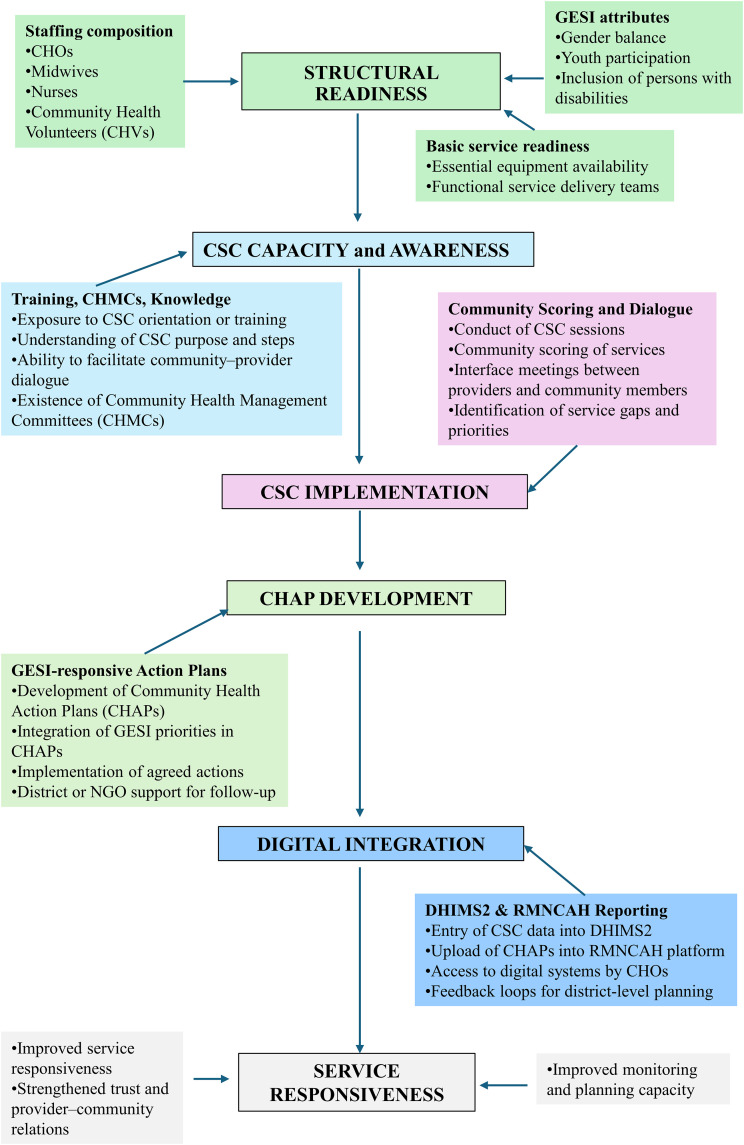
Conceptual framework linking CHPS staffing, community scorecard implementation, digital integration, and service quality within the Q4H baseline context.

The data in this manuscript derived from the baseline assessment conducted under the USAID Quality Services for Health (Q4H) Activity in collaboration with the Ministry of Health and its agencies such as the district-level leadership. The district-level leadership plays an important role in CHPS implementation. All districts operate under the oversight of a single Regional Health Directorate, which provides strategic direction and coordination. The baseline assessment was carried out before the implementation of Q4H interventions aimed at improving the availability and quality of health services in the public and private sectors. The Activity focused on strengthening service delivery for poor and vulnerable populations in six districts in northern Ghana. Q4H sought to build institutional capacity at national, regional, and district levels to enhance leadership, governance, and quality improvement systems. It also aimed to support local teams to identify and address systemic or site-level quality challenges through improved data use. In addition, the program worked to strengthen CHMCs to coordinate and promote a culture of quality. The following conceptual framework (Fig 1) shows the design of the project.

### Study design

This study adopted a cross-sectional design to examine the existence, staff category, operations, and functionality of CHPS across 86 zones in six districts. Data were collected from operational CHPS zones using quantitative and qualitative approaches, enabling an assessment of staff categorization, CSC training and utilization, CHPS functionality and the extent to which health facility results was uploaded into the District Health Information Management System II (DHIMS 2) and Community Health Action Plans was updated into the Reproductive, Maternal, Newborn, Child, and Adolescent Health (RMNCAH) platforms.

In this study, CHPS functionality was operationalized using a five-component framework grounded in national CHPS implementation guidelines [28]. A CHPS zone was classified as functional if it met all of the following criteria: (1) the presence of at least one assigned service-provision personnel (Community Health Officer or equivalent cadre); (2) an active Community Health Management Committee (CHMC); (3) availability of essential basic equipment required for routine primary care delivery; (4) provision of the core CHPS service package, including community outreach, home visits, management of minor ailments, antenatal care, and referral services; and (5) presence of formally recognized Community Health Volunteers (CHVs) supporting community-level service delivery. Zones failing to meet one or more criteria were categorized as partially functional or non-functional.

### Selection of CHPS zones

In all the six study districts, the research team worked in collaboration with the regional and district health authorities to identify operational CHPS zones. Selection was guided by the national criteria for an operational CHPS zone, including: (i) the presence of a resident Community Health Officer (CHO), (ii) an active CHPS compound providing routine service delivery, (iii) functional community health management structures (such as a CHMC), and (iv) evidence of regular outreach or community-based activities. Within each district, 13–15 zones were purposively selected, resulting in a total of 86 CHPS zones: 13 from Gushegu, 14 each from Mion and Nanton, and 15 each from Yendi, Sagnarigu, and Karaga. The CHPS linked to these zones constituted the study sample.

### Quantitative methods

Quantitative data were obtained through survey conducted using the KoboCollect [29] mobile application on handheld tablets. Based on CHPS zone records across the study districts, interviews were planned with 86 community health workers (CHOs and other health staff). Semi-structured questionnaires were designed specifically for this study to address the information gaps identified in the literature. This tool was then programmed into KoboCollect [29] to enable electronic data capture and secure transmission of completed interviews to a central database once internet connectivity was available. The tool covered six sections aligned with the study objectives: (i) sociodemographic information, (ii) CHPS staff categorization, (iii) functionality and routine activities, (iv) staff training on CSC, (v) the development of CHAPs, and (vi) dissemination of CSC/CHAP outputs into digital health platforms, including DHIMS2 and the RMNCAH reporting system. Tool development involved reviewing existing literature on CHPS monitoring and accountability frameworks. All interviews were conducted at participants’ respective workplaces.

### Qualitative methods

The qualitative component of the study combined key informant interviews with direct observations using a structured checklist designed before data collection. Research questions were designed to examine multiple dimensions, including CHPS staff categorization, functionality, staff training on CSC, the development of CHAPs, and their dissemination through digital health platforms. Participants were purposively selected to represent diverse social groups within each district. One interview was conducted in each of the 86 sampled CHPS zones. This deliberate selection approach ensured the inclusion of varied perspectives across social groups, thereby enhancing the participatory nature of the study.

### Study population and study sample

The study sample comprised Community Health Officers (CHOs) or other health staff (Community Health Nurses or Enrolled Nurses) affiliated with CHPS. To assess CHPS staff categorization, functionality, CSC training, CHAPs development, and their dissemination through digital platforms, one CHO was targeted to be interviewed per CHPS zone. In instances where a CHO was unavailable or not assigned to a zone during the data collection, another health worker (a Community Health Nurse or Enrolled Nurse) was designated as a replacement. A total of 86 CHOs or health workers were interviewed

### Data collection procedure and quality controls

Seventeen individuals formed the data collection team, including twelve research assistants and three supervisors from the study districts and two mobile data trainers responsible for programming the tools. Selection criteria included academic qualifications, prior engagement with district health systems, familiarity with CHPS zones, proficiency in English Language, and knowledge of local community dynamics. Training was held from March 13–15, 2024, at the Regional Health Directorate Training Unit in Tamale, covering field protocols, ethical procedures, structured observation, tablet-based data collection, and adherence to interview guidelines. The instruments were pretested with 5 respondents, and refined based on feedback. Data collection occurred from March 16–20, 2024, using mobile tablets that eliminated the need for manual entry and reduced transcription errors. Daily monitoring and real-time query resolution ensured data quality throughout the fieldwork.

### Statistical analysis

Quantitative data were exported from KoboCollect and analysed using R software version 4.5.0 [30]. The analysis relied on descriptive statistics because the study was designed as a baseline assessment rather than an intervention or hypothesis-testing study. The sample size (n = 86 CHPS zones) and the non-probabilistic selection of zones limited statistical power and violated assumptions required for reliable inferential analysis. Therefore descriptive statistics were employed to examine CHPS staff categorization, functionality, CSC staff training, CHAPs development, and dissemination through digital platforms. This is to establish an empirical reference point for subsequent intervention phases and follow-up evaluations. Results are presented as frequencies, proportions, means, ranges, and graphical summaries, disaggregated by district/municipality.

### Qualitative analytical methods

Qualitative data underwent an iterative thematic analysis. Responses to open-ended questions collected through KoboCollect were compiled in Excel, where the research team first familiarized themselves with the dataset. An initial coding framework was developed deductively, informed by the study objectives, the quantitative assessment, and literature on community health systems, gender norms, and primary healthcare governance [31,32]. A coding framework was developed to capture key concepts, patterns, and relationships within the data. This initial framework provided a structured starting point for organizing the qualitative material. Coding was then conducted collaboratively by the research team, who iteratively refined and expanded the framework to incorporate inductive codes emerging from participants’ narratives [31]. Intercoder reliability checks were performed to ensure consistency among analysts. Codes were organized into broader themes and subthemes. Attention was given to the interplay of gender norms, social roles, cultural beliefs, and community practices in shaping power dynamics that influence access, participation, decision-making, inclusion, exclusion, and vulnerability in health outcomes. Team reflections and joint discussions were employed to interpret findings, triangulate perspectives, and enhance the credibility and trustworthiness of the analysis.

### Ethics consideration

Ethical approval for this research was granted by the University for Development Studies Institutional Review Board (UDS/RB/024/24). Participants were thoroughly briefed on the study’s objectives, anticipated benefits, and any potential risks or discomforts associated with participation. Engagement in the study was entirely voluntary, with participants explicitly informed of their right to withdraw at any point without incurring any penalties or adverse consequences. Written informed consent was obtained from all participants prior to the commencement of data collection. Strict measures were implemented to ensure confidentiality and anonymity; personal identifiers were replaced with unique codes during data entry and analysis. Data were securely stored, with access limited exclusively to the principal investigator and data analyst. In all dissemination of results, care was taken to ensure that no information capable of directly or indirectly identifying individual respondents was revealed.

## Results

### Socio-demographic characteristics of community health workers from study districts in northern region of Ghana

The socio-demographic characteristics of the community health workers (Table 1) revealed that majority (67.4%) were other health staff (Community Health Nurses (CHN) or Enrolled Nurses) and sex distribution were nearly balanced (51.2% females and 48.8% male). In terms of age distribution, about half of the respondents (51.2%) were younger than 35 years. All respondents (100.0%) reported having attained tertiary-level education. Concerning marital status, most respondent were were married (80.2%) and majority of the respondents were muslim religion (89.5%). The Dagomba ethnic group constituted the majority (77.9%) of the respondents, with majority (83.7%) residing in rural areas.

**Table 1:** Socio-demographic characteristics of community health workers in districts.

Variables	Frequency (%)
**Category of respondents**	
CHO	28 (32.6%)
Other health Staff (CHN or Enrolled Nurses)	58 (67.4%)
**Sex of respondents**	
Male	42 (48.8%)
Female	44 (51.2%)
**Age of respondents**	
<29	22 (25.6%)
30-35	22 (25.6%)
36-40	18 (20.9%)
>40	24 (27.9%)
**Educational level**	
Tertiary	86(100.0%)
**Marital status**	
Single	13 (15.1%)
Married	69 (80.2%)
Widowed	4 (4.7%)
**Religious affiliation**	
Christian	9(10.5%)
Muslim	77 (89.5%)
**Ethnicity**	
Dagomba	67 (77.9%)
Frafa	5 (5.8%)
Mamprusi	4 (4.7%)
Other (Kusasi, Bimoba, Dagari, Akan, Gonja, Kotokoli, Ga, Moshi)	10 (11.8%)
**Type of community**	
Rural	72 (83.7%)
Urban	9 (10.5%)
Peri-urban	5 (5.8%)

### Estimated CHPS zones staff and their categorization in the study districts

The staff of CHPS zones across the six districts in northern region of Ghana (Table 2) varied considerably. On average, CHPS zones had 6.4 staff, ranging from a minimum of 1 to a maximum of 24 staff per zone. Notably, at the district level, Nanton recorded the highest mean staff across its zones (12.1), followed by Sagnarigu (9.2), while Karaga reported the lowest (2.1). Gender distribution showed a higher average number of male staff (mean = 2.9) compared to females (mean = 3.4). However, this varied by district: Nanton (7.3) and Sagnarigu (6.8) reported more female staff than males, while Gushegu and Karaga remained male-dominated. Regarding staff categories, enrolled nurses (mean = 2.1, range = 0–13) were the most common staff across districts, followed by community health nurses (mean = 1.7, range = 0–4). Midwives were less represented overall (mean = 1.0, range = 0–5), though Sagnarigu (2.6) and Nanton (1.1) recorded comparatively higher averages. Registered nurses were also few in number (mean = 0.9, range = 0–4). Other specialized staff, including nutrition officers, disease control officers, and laboratory technicians, were observed in some districts, with the highest averages in Yendi (2.0) and Sagnarigu (1.8).

**Table 2:** Summary of estimated CHPS Zone staff and their categorization across six districts.

Variables	# of staff	Gushegu (n = 13)	# of staff	Karaga (n = 15)	# of staff	Mion (n = 14)	# of staff	Nanton (n = 14)	# of staff	Sagnarigu (n = 15)	# of staff	Yendi (n = 15)	Total staff (All districts)	All districts (n = 86)
**CHPS staff and sex**														
Total number of Staff: mean (Range)	68	5.2 (1, 9)	31	2.1 (1,5)	72	5.1 (1, 11)	170	12.1 (1, 24)	138	9.2 (2, 18)	69	4.6 (1, 15)	549	6.4 (1, 24)
Number of Male: mean (Range)	49	3.8 (0,6)	14	0.9 (0, 3)	41	2.9 (0, 5)	67	4.8 (0, 13)	36	2.4 (0, 6)	45	3.0 (0, 10)	252	2.9 (0, 13)
Number of Female: mean (Range)	19	1.4 (0, 4)	17	1.1 (0, 3)	31	2.2 (0, 6)	103	7.3 (0, 17)	102	6.8 (2, 16)	25	1.6 (0, 7)	297	3.4 (0, 17)
**Staff category**														
Community Health Officers	5	0.5 (0, 1)	3	0.3 (0, 2)	7	0.9 (0, 2)	5	1.1 (0, 2)	4	2.6 (0, 5)	4	0.5 (0, 2)	28	1.0 (0, 5)
Midwives	7	1.9 (0, 5)	4	0.07 (0, 1)	12	2.4 (0, 5)	16	5.4 (0, 13)	39	1.9 (0, 6)	8	1.3 (0, 5)	86	2.1 (0, 13)
Enrolled Nurse	25	0.8 (0, 2)	1	0.2 (0, 2)	33	0.6 (0, 2)	75	1.7 (0, 4)	29	1.2 (0, 2)	20	0.9 (0, 2)	183	0.9 (0, 4)
Registered Nurses	10	1.4 (0, 3)	3	1.3 (0, 2)	9	1.4 (1, 2)	24	2.8 (1, 4)	18	2.1 (1, 3)	13	1.3 (0, 3)	77	1.7 (0, 4)
Community Health Nurses	18	1.0 (0, 1)	19	1.0 (0, 1)	19	0.0 (0, 0)	39	1.0 (0, 1)	32	1.8 (1, 3)	19	2.0 (1, 4)	146	1.3 (0, 4)
Other staff (Nutrition, Disease control, Paediatric nurse, Physiatrist Assistant, laboratory technician)	4	1.0 (0, 1)	2	1.0 (0, 1)	0	0.0 (0, 0)	8	1.0 (0, 1)	9	1.8 (0, 3)	6	2.0 (0, 4)	29	1.3 (0, 4)

**# = difference.**

Interviews with CHOs and other staff revealed that the distribution, and staffing levels of community health workers within the CHPS zones were perceived as uneven with several zones reporting insufficient numbers of staff to meet service delivery demands. Participants highlighted challenges related to understaffing, particularly in remote districts such as Karaga which had the lowest mean staff and Gushegu, where the limited number of health workers was described as a major barrier to effective service delivery. In these areas, respondents emphasized long waiting times and the inability of health staff to conduct regular outreach due to workload. Staff shortages were also noted in districts such as Yendi and Mion, whose mean staffing levels (4.6 and 5.1 respectively) were higher than Karaga but still insufficient for meeting the population’s service needs. In districts such as Nanton and Sagnarigu, where staff numbers were relatively higher (Quantitative data), respondents noted that service availability was broader and more regular. Female staff presence in these districts was especially valued, with CHPS staff explaining that women felt more comfortable seeking maternal and child health services from female health workers.

*‘When we have more female nurses, the women in the community are encouraged to go for antenatal care and delivery at the CHPS compound.’ (***female health staff, Nanton**)

The interviews also underscored the importance of staff diversity. In districts such as Yendi and Sagnarigu, the presence of specialized staff (e.g., nutrition officers, disease control officers, and laboratory technicians) was perceived to enhance the scope of services beyond routine maternal and child health. However, respondents noted that such specialized staff were often not permanently stationed, limiting their consistent contribution to service delivery. Across districts, the shortage of midwives was repeatedly highlighted as a pressing gap. Respondents linked this to the continued reliance on traditional birth attendants (TBAs) in some areas, with health staff expressing concern about the associated risks.

*“We are only two staff here, and without a midwife, it is difficult to handle deliveries. Sometimes women still go to TBAs when they cannot reach us.”* (**Male CHO, Gushegu)**.

### Assessment of CHPS zones functionality

The functionality of CHPS zones varied across the six districts in northern region of Ghana based on a five-point indicator including availability of assigned health personnel, CHMCs, CHVs, basic equipment and provision of basic services (Table 3). It was found that all 86 CHIPS zones reported having assigned service provision personnel, community health management committees, and formal community health volunteers. The provision of basic equipment by the Ghana Health Service slightly varied in Karaga (66.7%) and Yendi (80.0%), yielding an overall availability rate of 87.6%. This variation across districts may reflects differences in district level procurement cycles, logistics management capacity, budgetary constraints Similarly, the delivery of core CHPS services (community outreach, home visits, treatment of minor illnesses, antenatal care, and referrals) was widely reported (92.5% overall). Consequently, overall CHPS functionality was estimated at 96.0%, ranging from 89.1% in Karaga to 99.4% in Gushegu. The study also revealed important gender and inclusion patterns in the composition of the formal community health volunteers (CHVs).

**Table 3:** Availability of personnel, equipment, and services, of functional CHPS zone across six districts.

Variables	Gushegu (n = 13)	Karaga (n = 15)	Mion (n = 14)	Nanton (n = 14)	Sagnarigu (n = 15)	Yendi (n = 15)	All districts (n = 86)
Assigned service provision personnel: Yes	100%	100%	100%	100%	100%	100%	100%
Availability of CHMC: Yes	100%	100%	100%	100%	100%	100%	100%
Availability of basic equipment: Yes	100%	66.7%	92.9%	85.7%	100%	80.0%	87.6%
Provision of basic services at CHPS zone (Community outreach, Home Visit, Treatment to minor illness, ANC, Referrals): Yes	97.0%	78.6%	97.2%	95.7%	95.0%	92.0%	92.5%
Availability of formal Community Health Volunteers (CHV): Yes	100%	100%	100%	100%	100%	100%	100%
Overall CHPS zone functionality (Yes to the above 5 indicators)	99.4%	89.1%	98.0%	96.2%	99.0%	94.4%	96.0%
Number of male formal CHVs	142	55	102	53	56	117	525
Number of female formal CHVs	51	15	26	9	31	50	182
Formal CHVs being persons with disabilities	0	1	0	1	0	6	8
Formal CHVs being Youth below 35 years	44	18	20	5	26	17	130
Formal CHVs being from minority ethnic group (Fulbe etc)	5	1	12	0	2	4	24

**Fig 2:**
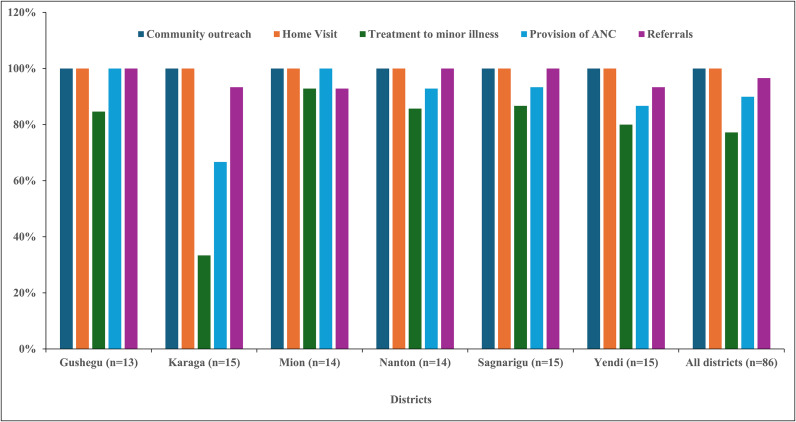
Provision of basic health services at CHPS zones in the six districts.

The provision of basic health services was generally high across all districts (Fig 2). The study showed that community outreach and home visits were universally implemented (100%). Similarly, referral services were widely available (97% overall), with full coverage in Gushegu, Nanton, and Sagnarigu, but with lower rates in Karaga and Yendi (93.3%). The treatment of minor illnesses showed variability across districts, with an overall coverage of 77%. Concerning the provision of antenatal care (ANC) services was widely reported (90% overall), though disparities persisted with Yendi (86.7%) and Karaga (66.7%).

The findings show high CHPS functionality but underscore critical equity gaps in equipment availability, service delivery, and inclusion. Interviews emphasized that CHPS zones were generally functional and fulfilled their expected roles in primary health care delivery. Respondents noted that the presence of assigned health personnel, community structures (CHMCs and CHVs), and the provision of basic services had built strong community trust in CHPS.

*‘The CHPS compound is now the first point of care. People rely on it because the staff are always available and the volunteers help us reach the community.’* (**Female CHO, Yendi**)

Despite this overall positive outlook, participants drew attention to variation in equipment availability and service coverage. In Karaga, CHPS staff reported that the lack of basic diagnostic equipment and delivery tools undermined service quality, particularly in managing maternal health and referrals. Similarly, some CHOs highlighted that inconsistent supply of essential drugs and equipment constrained the ability of CHPS zones to function effectively, though staff were present. Across the districts, respondents acknowledged that the predominance of male volunteers shaped the way outreach services were delivered. Women in particular noted the importance of female volunteers for services related to maternal and child health, as male volunteers were sometimes perceived as less approachable for sensitive health issues.

*‘When women are part of the volunteer team, it encourages more mothers to attend ANC and talk about family planning.’* **(female CHO, Mion)**

However, the overall low representation of women was attributed to cultural barriers and competing household responsibilities. The limited participation of youth, minority groups, and persons with disabilities was also raised as a concern. Respondents indicated that youth participants were rarely invited to join volunteer structures, and when included, they often felt their contributions were undervalued. Similarly, persons with disabilities were described as “clients” rather than “active participants” in CHPS service delivery.

*“We try to involve everyone, but often the same people are selected as volunteers, and young people or minority groups are left out.”* **(Male CHO, Mion)**

### Community health workers training on CSC and GESI-responsive CHAPs

**Fig 3:**
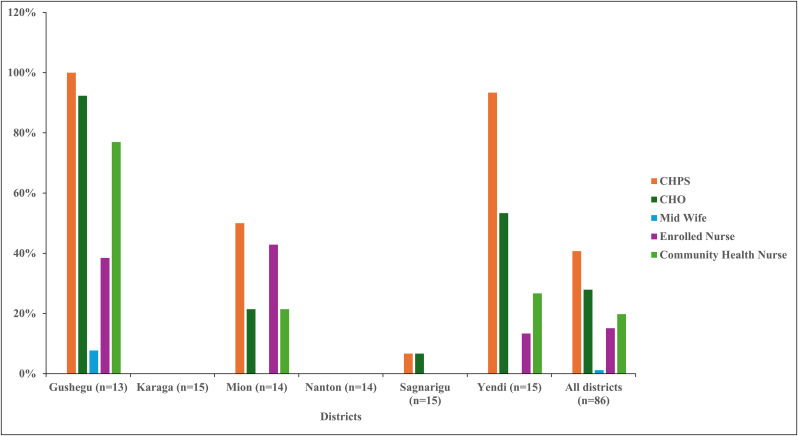
Training of CHPS staff in Ghana CSC in six districts.

The Fig 3 presents discrepancies in the training of community health workers on the utilization of the CSC assessments. While some districts show high levels of training coverage, others lag behind. For instance, in Gushegu district, all 13 CHPS zones have been trained, achieving full coverage of 100% and Yendi district exhibits a higher coverage of 93% of CHPS zones trained. Similarly, Mion district shows a moderate coverage of 50% with training. In Sagnarigu district, only 1 out of 15 CHPS zones have been trained. However, in Karaga and Nanton districts, no training has been reported in any CHPS zone. Looking at the numbers of staff trained, CHOs display varying levels of coverage across districts, ranging from 0% to 92%, with an average of 28% across all districts combined. On the other hand, midwives and enrolled nurses show minimal representation in terms of training across all districts. Community health nurses, however, demonstrate a wider range of coverage, from 21% to 77%, with an average of 20% across all districts combined.

**Fig 4:**
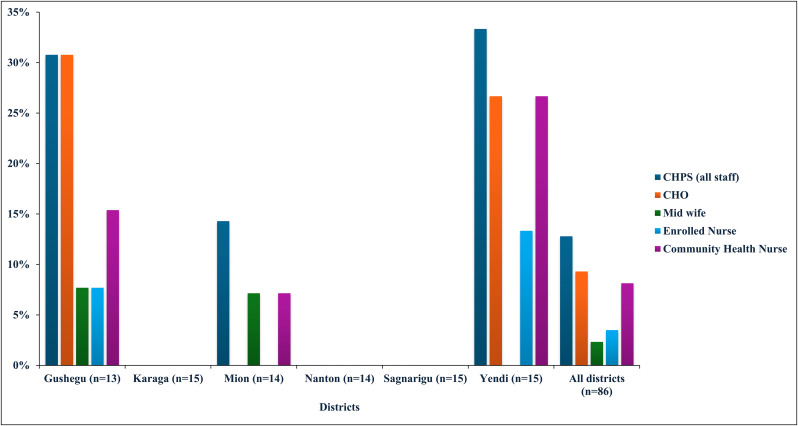
CHPS health staff trained to develop GESI-responsive CHAPs in six districts.

Fig 4 illustrates the extent of training received by community health workers on the development of GESI-responsive CHAPs, drawing on recommendations from Ghana’s CSC assessments in six districts of the Northern Region. Out of 86 CHPS zones assessed, only 11 reported that their staff had undergone such training. Yendi District accounted for the largest share, with 5 zones reporting trained personnel, whereas most other districts demonstrated minimal or no coverage. Across the trained staff, 8 CHOs, 2 midwives, 3 enrolled nurses, and 7 community health nurses were identified as beneficiaries.

The insights echo the quantitative results, showing that though some districts such as Gushegu and Yendi demonstrate promising models of capacity building, large sections of CHPS zones remain undertrained. Interviews with community health workers highlighted a consistent recognition of the importance of training on the CSC and GESI-responsive planning. However, participants emphasized that training coverage varied, with some districts better resourced than others. In Gushegu, where full coverage was achieved, respondents attributed this success to strong district-level leadership and external support from Non-Governmental Organizations (NGOs).

*‘We were all trained together, and that has made our work easier. We know how to collect feedback and include the community in planning.’* **(Male CHO, Gushegu)**

In contrast, Karaga and Nanton, where no training had been delivered, CHOs expressed challenges over their inability to apply CSC assessments in their work.

*‘We hear about the CSC, but we have never been trained. We are expected to use it, but we don’t know how.’* **(Male Health staff, Karaga)**

Similarly, CHMC members in Nanton voiced concerns that the lack of trained staff weakened accountability, leaving community voices underrepresented in CHPS decision-making processes. Even in districts with moderate training coverage, such as Mion (50%) and Sagnarigu (7%), stakeholders reported challenges in ensuring consistent and effective use of the CSC. Health officers explained that partial training limited knowledge transfer within teams, as only a small proportion of CHOs or nurses had been trained. This often led to “knowledge bottlenecks,” where implementation depended on one or two trained individuals rather than being a collective practice.

The findings on GESI-responsive CHAPs training further underscored capacity gaps. Community health workers admitted that GESI considerations were rarely integrated into planning.

*‘The training opened our eyes to how to include women, youth, and vulnerable groups in our health plans. But without refresher training and support, it is hard to sustain.’* **(Male CHO, Yendi)**

In districts with no exposure to such training, respondents noted that planning often overlooked the needs of women, youth, and persons with disabilities. Health workers also pointed to staff categories most often excluded from training. Midwives and enrolled nurses were rarely trained, despite their central role in maternal and child health service delivery.

*‘We work directly with mothers every day, but no one has trained us on CSC or GESI. It feels like these trainings are only for CHOs.’* **(Female Health staff, Sagnarigu)**

This exclusion creates challenges and a sense of disconnect between different CHPS staffs.

### CHPS service delivery improvements based on CSC recommendations

Table 4 summarizes district-level implementation of CSC recommendations, staff training, and feedback mechanisms within CHPS zones. Overall, fewer than half of the CHPS zones (46.5%) out of 57 CHPS zones reported implementing service delivery improvements based on CSC recommendations. However, there were stark inter-district differences: Yendi (80.0%), Sagnarigu (73.3%), and Mion (64.3%) demonstrated higher levels of implementation, while Karaga and Nanton reported no improvements linked to CSC recommendations. Feedback mechanisms showed a stronger presence. Across all districts, 58.3% of CHPS zones reported conducting joint feedback sessions involving DHMT, CHPS staff, CHMC members, and community representatives. Karaga (100%) and Nanton (85.7%) achieved the highest coverage, while Sagnarigu had the lowest (13.3%). Regarding frequency, 47.8% of zones reported quarterly feedback sessions, 6.1% reported biannual sessions, and 4.5% reported annual sessions. Community engagement feedback sessions conducted specifically by CHMCs were observed in 56.7% of CHPS zones. Coverage was highest in Mion (85.7%) and Sagnarigu (86.7%), while Nanton (14.3%) reported the lowest level of such engagement.

**Table 4:** Summary of the district-level implementation of CSC recommendations, training, and feedback within CHPS zones in six districts.

Variables	Gushegu (n = 13)	Karaga (n = 15)	Mion (n = 14)	Nanton (n = 14)	Sagnarigu (n = 15)	Yendi (n = 15)	All districts (n = 86)
CHPS zone implemented service delivery improvements based on CSC recommendations: Yes	61.5%	0.0%	64.3%	0.0%	73.3%	80.0%	46.5% (n = 57)
Feedback sessions involving DHMT, CHPS staff, CHMC members and community members on the status of CHAPs implementation: Yes	53.8%	100.0%	57.1%	85.7%	13.3%	40.0%	58.3%
**Frequency of feedback**							
*Quarterly*	30.8%	60.0%	57.1%	85.7%	13.3%	40.0%	47.8%
*Biannually*	23.1%	13.3%	0.0%	0.0%	0.0%	0.0%	6.1%
*Annually*	0.0%	26.7%	0.0%	0.0%	0.0%	0.0%	4.5%
Community engagement feedback sessions conducted by CHMC?: Yes	53.8%	46.7%	85.7%	14.3%	86.7%	53.3%	56.7%

Table 5 presents district-level service delivery improvements that were directly linked to CSC recommendations. The data highlight diverse forms of progress across districts, spanning infrastructure, service quality, staff performance, and community engagement. In Gushegu, reported improvements included enhanced skilled delivery, improved drug availability, better staff attitudes, strengthened anaemia management, and infrastructure upgrades such as renovation and construction of CHPS compounds. Additional gains were noted in Water, Sanitation and Hygiene (WASH) services, maternal and child health, malaria control, and the provision of a shade structure for staff and clients. In Mion, improvements were strongly community-driven, including the construction of a maternity block, provision and maintenance of staff accommodation, and support for skilled delivery. Service delivery was further strengthened through increased early ANC registration, improved referral systems, and greater involvement of traditional birth attendants (TBAs) in referring pregnant women to CHPS facilities (physical primary health-care facility located within a CHPS zone). In Sagnarigu, enhancements focused on service access, quality, and facility readiness. Specific gains included extension of piped water to CHPS facilities, improved drug availability, reduced waiting times through staff shift systems, and early staff reporting. Community health volunteers were engaged in home visits, while facility infrastructure was improved through the construction of washrooms and enhanced cleaning routines. In Yendi, the scope of improvements was broader and more comprehensive. Key areas included malaria control, improved drug availability, and strengthened staff attitudes. Services expanded to encompass family planning, ANC, postnatal care (PNC), and child welfare clinic (CWC) activities. Additional community-driven contributions included mobilization of funds for temporary health facilities, improved home visits, facility cleaning and safety measures, and better utilization of the National Health Insurance Scheme (NHIS).

**Table 5:** District-level reported service improvements linked to the implementation of CSC recommendations across CHPS zones in the selected northern region of Ghana.

Improved service delivery based on CSC recommendations	Services improved
**Gushegu**	• Skilled delivery, • Availability of drugs, • Staff attitude improvement, • Anaemia management, • Renovation and construction of CHPS compound, • WASH, maternal and child health, malaria services, • Construction of a shade for staff/clients
Mion	• Community initiated construction of a Maternity Block for enhanced MCH services, • Provision of accommodation for health staff and maintenance of the health facility, • Skilled delivery, • Community provision of staff accommodation through CSC recommendations, • Improved early ANC registration and attendance, • Improvement in skilled delivery, • Increased referral of pregnant women by TBAs to deliver at CHPS facility
Sagnarigu	• Extension of pipe water to CHPS and community engagement on attendance, • Availability of drugs, • Reduction of waiting time at facilities through staff shifts, • Community health volunteers conducting home visits, • Staff reporting to work early, • Construction of washroom for clients and facility cleaning, • Skilled delivery, • Increased home visits
Yendi	• Malaria control (including provision of burning pit, washrooms, sea sand for blocks, chairs, tables), • Availability of drugs, • Staff attitude improvement, • Support for referrals and outreaches, • Family planning, ANC, PNC, CWC services, • Under-five malaria improvement, • Mobilisation of funds for temporary health facility and weekly home visits, • Improved NHIS utilisation, • Safety and cleaning of facilities, • Improved home visits by CHMCs

Results confirmed that the adoption of CSC recommendations varied across districts, reflecting differences in leadership, community participation, and resource availability. Respondents from Yendi, Sagnarigu, and Mion highlighted how CSC assessments had catalysed significant improvements in service delivery.

*‘After the community scorecard, we saw changes, more attention to family planning, antenatal care, and malaria control. The community also contributed funds to improve facilities, which motivated us as staff.’* **(Male CHO, Yendi)**.

These accounts align with the higher quantitative coverage of CSC-linked improvements (80.0% in Yendi, 73.3% in Sagnarigu, and 64.3% in Mion). In contrast, participants in Karaga and Nanton, where no CSC-driven improvements were reported, described a sense of disconnect between assessments and actual service changes.

*‘We attend meetings and hear about the CSC, but nothing changes in our CHPS. There is no follow-up, no training, and no resources to act.’* **(Male health staff, Karaga)**

This gap reveals the structural and resource challenges that limit translation of community feedback into action. The role of community contributions emerged strongly. In Mion, a health worker described how residents mobilized resources to build a maternity block and support staff housing, thereby directly addressing service gaps identified through the CSC. Similarly, Gushegu reported infrastructure renovation, improved WASH services, and strengthened maternal and child health services.

*‘The CSC made it clear what we were lacking, and the community took it upon itself to act. Even small changes like building shade structures made a difference for staff and patients.’* **(Male CHO, Gushegu)**

Taken together, the qualitative evidence reinforces the quantitative findings: while CSC processes have stimulated notable improvements in some districts, gaps persist in some where improvements remain absent. Moreover, the sustainability of changes depends heavily on community resource mobilization and the functionality of feedback platforms.

*‘The CSC can bring change, but only if there is leadership, community commitment, and follow-up support. Otherwise, it remains just a discussion tool.’* **(Male CHO, Mion)**

### Uploading of CSC results and updating of CHAPs into the digital platforms

#### District Health Information Management System II (DHIMS 2).

**Fig 5:**
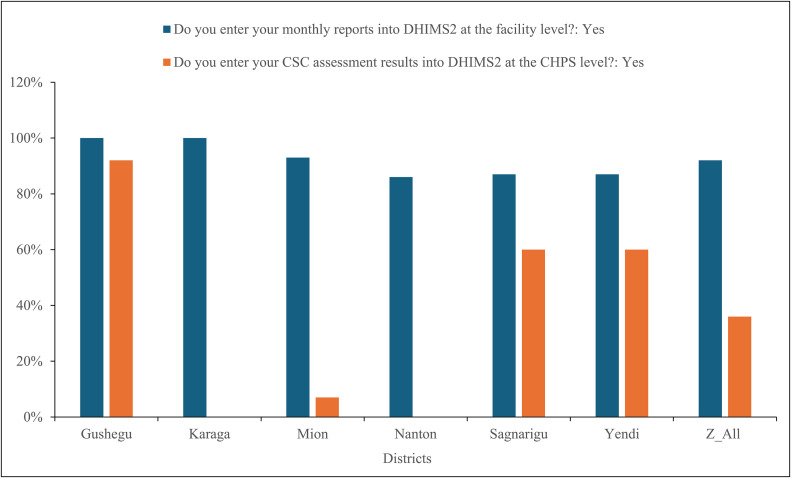
Enter monthly reports into DHIMS2 at health facility level in six districts.

Fig 5 presents findings on the capacity of CHOs to enter data from the Ghana CSC quarterly assessments into the DHIMS 2 at the CHPS level. Concerning the routine monthly reporting, all districts except Nanton district demonstrated high levels of compliance, ranging between 86% and 100%. Nanton district recorded the lowest rate at 86%. However, reporting on CSC assessment results into DHIMS 2 at the CHPS level revealed major inter-district variability. Gushegu district achieved the highest rate of entry at 92%, followed by Sagnarigu and Yendi Municipals, each reporting 60%. In contrast, Karaga and Nanton districts reported no entry of CSC assessment data into the system.

#### Reproductive, maternal, newborn, child and adolescent health.

**Fig 6:**
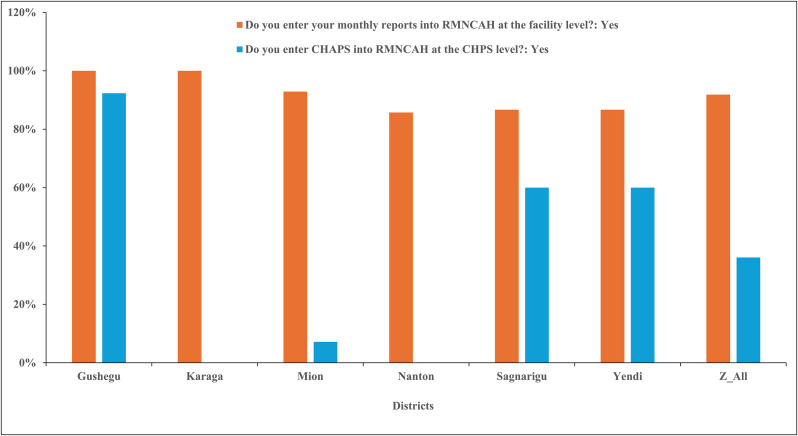
Enter CHAPs into RMNCAH at health facility level in six districts.

Fig 6 presents findings on the capacity of CHOs to enter CHAPs into the Reproductive, Maternal, Newborn, Child and Adolescent Health (RMNCAH) platform at CHPS level. At the facility level, all districts with the exception of Nanton district reported high compliance in entering quarterly CHAPs into RMNCAH, with rates ranging from 86% to 100%. Nanton district recorded the lowest compliance at 86%. In contrast, the entry of CHAPs into the RMNCAH platform at the CHPS level showed notable inter-district variability. Gushegu district demonstrated the highest compliance at 92%, followed by Sagnarigu and Yendi Municipals, each reporting 60%. In comparison, Karaga and Nanton districts reported no entry of CHAPs into the RMNCAH system.

### Challenges with DHIMS2 and RMNCAH digital platforms

The interviews with community health workers revealed several structural and operational barriers that explain the varied reporting of CSC results into DHIMS2 and the limited updating of CHAPs into the RMNCAH platform. A cross-cutting theme across the districts was a lack of technical capacity and inadequate training support.

‘*We were trained to collect the CSC data, but not all of us know how to upload it into the system. Sometimes we just send the paper reports to the sub-district and that ends it’* **(Male CHO, Mion).**

‘*We depend on one or two staff who know how to use the computer. If that person is not around, nothing goes into DHIMS2’* **(Male health staff, Karaga).**

Additionally, to knowledge gaps, staff pointed to logistical constraints such as unreliable internet connectivity, limited access to computers, lack of access to the platforms and administrative bottlenecks within the health directorates.

‘*The network is often slow here too. Even when we try to enter the CHAPs into RMNCAH, the system does not respond, so people give up and wait for the district office’* (**Female Health Staff, Sagnarigu).**

*‘The platform is controlled from the district level. At the CHPS compound, we don’t have the login details, so we cannot do the entry ourselves’* **(Female health staff, Karaga).**

‘*Some of the reports are submitted on time, but because of internal procedures, they are not uploaded into DHIMS2 immediately. That creates gaps in the system’* **(Male CHO, Karaga).**

## Discussion

This study set out to assess staff mix and functionality of CHPS zones in Northern region of Ghana, as well as their ability to use the Ghana CSC to evaluate health facility performance, develop CHAPs and disseminate these through existing digital platforms. Our findings indicate that although CHPS zones are largely functional and provide widespread service coverage, disparities in staffing, uneven training, gender and social inclusion gaps, and limited follow-through on CSC recommendations continue to undermine equitable and sustainable community health service delivery. The qualitative evidence highlighted systemic barriers, including limited technical capacity, inadequate digital literacy, insufficient infrastructure, and weak district-level coordination. The findings provide context-specific evidence to inform policy and practice aimed at strengthening PHC delivery and accelerate progress towards UHC.

### CHPS zones staff mix and functionality

Equitable distribution of skilled and gender equity staff is essential for strengthening community health systems. Interpretation of staffing adequacy requires situating the observed numbers against national norms. Ghana’s CHPS Operational Policy Framework recommends at least one CHO supported by Community Health Volunteers for each CHPS zone, with two or more CHOs required in high-population catchments exceeding 3500 residents. However, recent studies report that many CHPS zones operate below these standards, with staffing deficits exceeding 30–40% in rural districts [14,33]. This contextualizes respondents’ perceptions of workload pressure, long waiting times, and challenges in sustaining outreach activities, particularly in Karaga, Yendi, and Mion. The study also revealed marked differences across districts.The uneven presence of specialized cadres, such as nutritionists, disease control officers, and midwives, mirrors wider trends across Ghana’s health workforce, where persistent vacancies and inequitable deployment, estimated at 41% in some cadres, remain a challenge, especially in rural and deprived areas [4,34]. However, health worker attraction and retention in rural and peri-urban areas is influenced by multiple health systems and contextual factors. Studies from Ghana and similar settings show that proximity to basic social amenities (schools, electricity, potable water), better working conditions, and rural background of staff are associated with longer tenure and higher retention [35]. Districts such as Nanton and Sagnarigu may have benefited from higher levels of urbanicity, better road connectivity, and closer proximity to regional administrative centres. Health workers often cite poor infrastructure, inadequate equipment, and limited career progression opportunities as barriers to remaining in remote postings, while access to resources and supportive work environments improve retention. In Ghana, rural and remote districts with better road access and social services may offer relatively more favourable conditions that support staff retention, while strong local leadership and managerial support can further motivate staff by fostering professional recognition and workplace satisfaction [35,36].

Evidence from sub-Saharan Africa confirms that health worker maldistribution undermines service quality and equity. In Tanzania and Malawi, rural facilities have been shown to be disproportionately under-resourced, with adverse consequences for maternal and child health [37,38]. Our observation that districts with higher proportion of female staff experienced greater maternal health service uptake aligns with regional and global findings: women’s comfort and trust in antenatal and delivery care increases when services are provided by female health workers [10,39,40]. A meta-analysis across 33 sub-Saharan African countries similarly showed that the presence of female providers significantly improved antenatal coverage and skilled birth attendance [41].

The limited number of midwives in our study reflects wider shortages across Ghana and contributes to continuous reliance on traditional birth attendants (TBAs), with associated maternal risks [42]. Comparable difficulties have been noted in Nigeria, where the Midwives Service Scheme struggled with retention and sustainability [43]. These results reinforce WHO’s argument that an equitably distributed, multidisciplinary primary health care workforce is indispensable for achieving universal health coverage [44,45].

While overall functionality of CHPS zones was high, with near universal coverage of core services such as home visits and referrals, gaps in equipment provision mirror wider weaknesses in supply chain management across LMICs, where staff are often present but unable to function effectively without basic diagnostic tools [46,47]. The male predominance among CHVs across districts highlights persistent sociocultural barriers that limit women’s participation in community health work, a phenomenon also documented in Tanzania and Sierra Leone [48,49]. Similarly, the limited inclusion of youth, minority ethnic groups, and persons with disabilities reflects entrenched stigma and marginalisation patterns seen elsewhere in Africa [17]. Although equitable inclusion of persons with disabilities is essential, the expectation of high participation as CHPS volunteers by the respondent may be unrealistic. Given that not all person with disability experience mobility or functional limitations. Thus, while CHPS zones provide essential services, persistent equity gaps remain, shaped by structural, cultural, and systemic constraints.

### CSC training and GESI-responsive CHAPs

Training coverage on the CSC and GESI-responsive CHAPs was highly variable. This unevenness echoes findings across Ghana and other LMICs, where training for community health workers is fragmented, reliant on external donor support, and rarely institutionalised [14,49]. Knowledge bottlenecks, where only a few staff are trained, mirror Ethiopia’s Health Extension Programme, where selective training limited sustainable practice [50].

Globally, systematic capacity-building for community health workers has been shown strengthen accountability, service delivery, and engagement [51,52]. The exclusion of midwives and nurses from CSC and GESI training reflects persistent professional hierarchies that undermine team-based PHC delivery [48,53]. Districts that performed better benefitted from strong leadership and NGO support, consistent with evidence that decentralised leadership and external facilitation shape outcomes in community health systems [54]. The near absence of GESI-focused training across most districts demonstrates how equity and social inclusion remain peripheral in practice despite policy rhetoric, echoing similar critiques from Sierra Leone and Liberia [49]. These results suggest that Ghana’s CHPS system reflects both the promise and pitfalls of community-based health reforms: while district-level leadership and NGO engagement can deliver model outcomes, sustainable and equitable training requires institutional embedding, regular refresher courses, and inclusion of all cadres of staff to prevent inequities and ensure collective accountability.

### Service delivery improvements based on CSC recommendations

The results on the implementation of CSC recommendations and associated service delivery implementation of CSC-driven recommendations were uneven. These results reflect findings from Uganda, Malawi, and Tanzania, where CSCs have improved accountability and health service delivery but only when supported by committed leadership, resources, and facilitation [8,55,56].

Feedback mechanisms were more common than actual service improvements, suggesting that while community engagement platforms are relatively easy to institutionalise, translating feedback into funded service changes remains a challenge, as observed in Ethiopia and Kenya [57,58]. Reported gains, such as improved skilled delivery, early ANC uptake, drug availability, and WASH services, are consistent with studies linking CSC processes to improved maternal and child health outcomes [7,59]. The variation observed across districts underscores the context-specific nature of CSC outcomes, heavily dependent on leadership and community mobilisation.

### Dissemination of CHAPs through digital platforms

Although routine reporting into DHIMS2 was strong across most districts, uploading of CSC results and CHAPs into DHIMS2 and RMNCAH was inconsistent. The inconsistent uploading of CSC results and CHAPs into DHIS2/RMNCAH reduces the visibility of community-generated indicators within routine decision-making processes and thus limits the ability of managers to respond to community-identified problems. This discrepancy between established reporting and newer participatory indicators is well documented in LMICs: community-generated data often face challenges of technical capacity, digital literacy, and access [60–62]. Similar difficulties have been reported in Nigeria and Tanzania, where reliance on paper-based submissions and lack of login access hindered digital reporting [63,64]. Empirical evidence showed that CSCs can change service processes and demand (for example, by reducing informal payments, improving provider-community dialogue, and increasing utilization). However, these effects are frequently measured and held locally unless deliberately integrated into national health reporting systems. In Kisumu (Kenya) and Ntcheu (Malawi), CSC interventions produced measurable improvements in community perceptions, provider behaviour and selected service outcomes. However, those improvements were tracked using targeted monitoring systems and local facility records rather than systematically captured in national HMIS dashboards [65,7]. By contrast, the DHIS2 platform functions as the canonical repository for routine service metrics used in planning and resource allocation. Studies demonstrated that while facility reporting into DHIS2 is often high in Ghana, the use of DHIS2 data for local planning and action especially at sub-district and community levels remains variable. This is constrained by training gaps, limited infrastructure, and weak data-use cultures. These structural barriers explain why community-level, participatory outputs (CSC/CHAPs) are less likely to be uploaded, validated, and used for iterative planning despite their local salience [66,67].

Bridging CSC processes with DHIS2/RMNCAH requires three interlinked actions. First, technical integration: define a minimal, standard metadata schema and data element set for CSC/CHAP outputs and expose these elements via the DHIS2 Scorecard/Tracker apps. This can also be done as community-level aggregate datapoints so they can be ingested and visualized alongside routine indicators. Second, capacity and workflow alignment: invest in data-entry access, training, and SOPs so facility and sub-district staff can routinely digitize validated CSC outputs without duplicative work. Studies have shown that local supervision and routine coaching markedly increase DHIS2 data entry and data use [66,68]. Third, governance and validation: institute clear governance rules for community-generated data (who approves, how action-points are validated, and how community and facility data are reconciled). This way community voice can inform district planning without compromising data quality or accountability. Evidence from CSC implementations underscores the need for negotiated validation processes where community and providers must jointly agree on indicators and verification steps for the data to be actionable and trusted [65,7]. Operationalizing these recommendations would increase the policy relevance of CSC outputs: when community-identified problems and CHAP actions are visible in the DHIS2/RMNCAH dashboards, district managers can allocate resources, schedule outreach, and track implementation as part of routine supervisory cycles. However, the literature warns that without parallel investments in connectivity, simplified reporting templates, and local data-use coaching, integration risks becoming an extra administrative task that is poorly completed. Thus, linking CSC to DHIS2 should be pursued as a systems intervention (technical configuration plus human capacity and governance reform) rather than a merely technical upload exercise [67].

Our qualitative data highlighted familiar bottlenecks: weak internet connectivity, lack of computers, limited staff skills, and restricted access to platforms. These barriers are not unique to Ghana; studies from Malawi, Mozambique and elsewhere have linked poor HIS performance to systemic resource gaps rather than unwillingness of staff [69–71]. Better-performing districts (Gushegu) benefitted from strong supervision and localised training, confirming that supportive leadership can partly mitigate structural challenges [72]. Currently, no internationally validated benchmarks exist for sub-national or facility-level integration of community-generated data into national health information systems. Existing digital health maturity frameworks (DHIS2 maturity profile, the WHO/PAHO Information Systems for Health Maturity Model, and the Global Digital Health Monitor) mainly assess system-level governance and infrastructure rather than operational performance at primary care facility or community-zone level. Therefore findings should be interpreted as within-system comparative performance across districts rather than against external numeric standards.

### Strengths and limitations

This study has important strengths. Its mixed-methods design, combining surveys, interviews, and observations across 86 CHPS zones in six districts, provides both breadth and depth. District-level disaggregation revealed meaningful variability often masked in national reports, and the explicit focus on the CSC–CHAP–digital integration pathway adds new insight into how accountability processes function in practice. Use of mobile data collection and real-time supervision improved data accuracy and reliability.

Limitations must be acknowledged. Purposive sampling limits generalisability, and reliance on self-reported data introduces the possibility of recall and social desirability bias. The short fieldwork duration constrained deeper exploration of governance processes at district level. The cross-sectional design prevents causal inference, and the involvement of local research assistants may have influenced responses despite measures to minimise bias. Nonetheless, these limitations do not detract from the contribution of providing contextually grounded, empirically rich evidence on CHPS functionality and social accountability. Additionally, this study did not include community members as qualitative respondents. Consequently, the qualitative findings reflect the perspectives of CHPS staff only, without incorporating community-level experiences or perceptions of CHPS functionality. This limits the ability to triangulate staff narratives with community viewpoints, which are integral to fully assessing service accessibility, quality, and responsiveness. Although structured direct observations were conducted using a predefined checklist, these data were excluded from the final analysis because the observational items overlapped substantially with information already captured through the quantitative survey. Including both would have resulted in redundancy without generating additional analytical value beyond what was already measured through interviewer-administered assessments of equipment availability, service provision and staffing. For this reason, only the survey-based measures were retained for analysis and reporting. Another limitation is that the absence of a quantitative GESI index and community-based focus group discussions limits the ability to statistically link inclusion dimensions with service uptake outcomes.

### Policy and practice implications

The findings demonstrate that the effectiveness of CHPS depends not only on the structural presence of facilities and staff, but also on equitable workforce distribution, sustained training, institutionalised accountability, and digital integration. Embedding GESI within staffing and governance processes is essential to ensure inclusivity. Scaling training beyond CHOs to all cadres, including midwives and nurses, will strengthen team-based care. District-level leadership and NGO partnerships remain crucial but should be complemented by institutional embedding to ensure sustainability. Finally, investments in digital infrastructure and local ICT capacity are required if community feedback is to be systematically incorporated into health information systems. Evidence from low- and middle-income settings suggests that targeted capacity building and digital support for community health workers can improve primary health care performance. A pilot intervention in rural Bangladesh demonstrated that training CHWs to use digital health tools increased the effectiveness of health promotion and screening activities over repeated follow-up periods [73]. This highlight the potential for improved service delivery when digital platforms are integrated with frontline worker practices. In sub-Saharan Africa, a review indicated that deeper integration of CHW programmes into national health systems including clear documentation processes and supportive supervision enhances CHW contributions to routine reporting and system responsiveness [74]. Moreover, lessons from digital health implementations across African settings show that adequate training, technological support, and tailored digital applications can strengthen CHW data reporting and decision-making, although challenges such as connectivity and infrastructure persist [75].

## Conclusion

This study provides new evidence on the staffing, functionality, and accountability mechanisms of CHPS zones in Northern region of Ghana. While CHPS zones were found to be broadly functional, persistent disparities in staff distribution, equipment availability, CSC training, and digital integration undermine their potential to deliver equitable and inclusive primary health care. The uneven implementation of CSC-driven recommendations and the limited integration of CHAPs into national health information systems reflect systemic gaps in capacity, coordination, and governance.

To maximise the transformative potential of CHPS, policies must be prioritise based on inclusive CSC training. This should be scaled beyond CHOs to include midwives, nurses, and district level supervisors. Training should be delivered through structured refresher sessions of sufficient duration to cover CSC facilitation, development of GESI-responsive CHAPs and basic use of digital health reporting tools. Additionally, workforce policies should prioritise equitable deployment and retention of multidisciplinary, gender-balanced teams. Moreover, investment in digital infrastructure and district-level ICT support should focus on integration CSC and CHAP outputs into existing platforms given the low levels of digital health systems use reported across CHPS zones. Embedding accountability and inclusion within CHPS operations is essential if community voices are to be translated into sustainable improvements in service delivery. Future research should adopt longitudinal and implementation-focused designs to explore how district leadership, capacity-building, and digital innovations interact to shape equity and sustainability in PHC delivery across LMICs.

## Supporting information

S1 File Minimal data 1. (XLSX)

S2 File Minimal data 2. (DOCX)
