# Supplementary material for: Making community-based health planning and services work: Staffing, accountability and digital integration for quality primary health care in Northern Ghana
Source: PLoS One. 2026 Feb 2;21(2):e0341176. doi: 10.1371/journal.pone.0341176 (PMC12863559; doi:10.1371/journal.pone.0341176)
Supplement: S2 File — (DOCX) [file pone.0341176.s002.docx]

**Background Information for respondents**

| **Guiding Question** | **Response from respondent** |
| --- | --- |
| How do you perceive the adequacy, composition, and distribution of staff within CHPS zones? | We are only two staff here, and without a midwife, it is difficult to handle deliveries. Sometimes women still go to TBAs when they cannot reach us. The pressure is enormous and most of the time, people wait long hours because we cannot attend to everyone at once. Without a midwife, we struggle to handle deliveries. The shortage is felt daily, and women often turn to TBAs when they cannot reach us. Outreach services also suffer because the workload at the compound leaves no room to step out. Male CHO, Gushegu |
|  | My role here is challenging because there are very few of us. Women prefer to see me for antenatal and child welfare services, but with the long queues, I cannot give each client the attention they need. The absence of a staff create huge gaps, and we end up referring many cases. This discourages women, who sometimes seek alternative care outside the facility. Male CHO, Gushegu |
|  | Every day, we try to manage with limited staff. Our community is very large, yet there are only a handful of us working here. Patients complain of delays, and during immunization campaigns, the workload becomes unmanageable. We lack the manpower to divide responsibilities properly. This has made many people feel that the facility is not reliable for quick services. Male CHO, Gushegu |
|  | I assist with record keeping and patient follow-up, but the shortage of professional staff makes my work harder. When CHOs are busy, I sometimes help explain health information to clients. Many women are discouraged by the absence of female midwives, and they prefer to give birth at home. We know this is risky, but we cannot cover all the needs with the current staff numbers. Male CHO, Gushegu |
|  | I am mainly responsible for mobilization, but even that becomes difficult when there are not enough health workers. I often have to calm patients who complain about delays. In my view, the staff here are too few for the size of the population. Outreach is limited, and many rural communities feel left behind because we cannot visit them regularly. Female CHO, Gushegu |
|  | The community depends heavily on the few staff we have, and as support staff, we try to fill in wherever possible. But it is not enough. Women sometimes come for services but leave without being attended because the CHOs are overwhelmed. This makes our health delivery system weaker and contributes to people’s mistrust of formal health services. Female Health Staff, Gushegu |
|  | I notice that the absence of specialized staff limits the services we provide. People often ask about nutrition or family planning, but without experts, we cannot fully respond. The shortage of midwives is the most serious issue here. Women in labor have no choice but to go elsewhere or use TBAs, and that places them at serious risk. Female Health Staff, Gushegu |
|  | My work here is mostly administrative, but I see how the shortage of health workers affects service delivery. The queues are always long, and people get frustrated. Many times, mothers leave without getting care because they cannot wait. This weakens the confidence the community has in us, but the truth is, with so few staff, we cannot do more. Female Health Staff, Gushegu |
|  | Our community is large, and the staff available are very few. Even when CHOs try their best, it is impossible to meet everyone’s needs. Outreach is rare, and when it happens, we have to suspend services at the compound. It is clear that unless staffing improves, our facility will continue to struggle. Female Health Staff, Gushegu |
|  | I have seen many women turn back home when they find out no midwife is available. They believe without midwives, they cannot safely deliver at the compound. This reality makes people rely on TBAs, which is dangerous. The staff here are doing their best, but there are just not enough of them to cover all the demands. Female Health Staff, Gushegu |
|  | The shortage of staff is affecting our capacity to maintain proper health records. With CHOs overwhelmed, record-keeping is rushed, and sometimes follow-ups are missed. This leads to gaps in service provision. I believe that adding more trained staff, especially midwives and nurses, would make a big difference for our community. Male Health Staff, Gushegu |
|  | Every clinic day, we are faced with too many patients compared to the number of staff available. Mothers waiting with babies often complain of the long hours, and some do not return for follow-ups. Without adequate staff, we are unable to provide the quality of care the community deserves. Male Health Staff, Gushegu |
|  | In my role, I see how overstretched our CHOs are. Sometimes patients have to be turned away, not because we do not want to serve them, but because the staff are simply exhausted. The absence of specialized staff such as midwives and nutrition officers makes the situation worse. The community feels the gap, and so do we. Male Health Staff, Gushegu |
|  | Staffing here in Mion is relatively better than in some districts. This helps us share responsibilities. Women here especially appreciate the presence of female nurses for antenatal and delivery services. The workload is still high, but at least we are able to conduct outreach and routine services more consistently than in Gushegu or Karaga. Male CHO, Mion |
|  | I feel supported working here because there are other colleagues to share the burden. This makes it easier to provide antenatal and child health services without leaving people unattended. However, during busy periods, such as immunization campaigns, the workload is still heavy. Transport for outreach remains a challenge even with relatively more staff. Male CHO, Mion |
|  | Our facility is functioning better because of the balance between male and female staff. This mix encourages women to attend services more confidently. Still, the shortage of midwives is a serious issue here. Without them, deliveries are not as safe, and many women remain hesitant to fully trust the facility. Male CHO, Mion |
|  | I am part of the support team, and I see the difference that a balanced number of staff makes. Services run smoother compared to other districts, and waiting times are not as long. The challenge remains with transport for outreach and the shortage of specialized staff such as midwives, which still limits the scope of what we can offer. Male CHO, Mion |
|  | Women in the community feel more confident coming to the facility because they know they will meet female nurses. This is very important for maternal and child health services. However, when specialized services like nutrition counseling are needed, we struggle because those staff are not permanently stationed here. Male CHO, Mion |
|  | With more staff here, outreach services are more regular, which helps reach rural communities. But fuel and transport are still inadequate, limiting how much we can cover. The absence of consistent midwives remains a problem. When emergencies occur, it is a real struggle for both staff and patients. Female CHO, Mion |
|  | I am responsible for health education and mobilization, and I find it easier to work with a larger team. But the lack of midwives is something the community complains about constantly. Women still depend on TBAs in emergencies, and that is risky for both mothers and babies. Female CHO, Mion |
|  | Staffing levels here are better, but still not sufficient for the size of the population. During health campaigns, we are overwhelmed despite having more CHOs compared to Gushegu. Specialized staff are helpful but not always available. This limits the overall quality of service delivery. Female CHO, Mion |
|  | My work with records is smoother because the workload is shared among more people. Patients here also appreciate the presence of female nurses. Still, the community continues to ask why there are no permanent midwives, because this affects their decision to deliver at the facility. Male Health Staff, Mion |
|  | I believe our staffing situation is manageable, but we are still far from adequate. People expect a wide range of services, but without permanent specialized staff, we cannot meet all their expectations. The presence of female CHOs makes a positive difference, especially for maternal health. Male Health Staff, Mion |
|  | I think, we are better off in Mion. Outreach is more regular, and waiting times are shorter. But without midwives, deliveries are still a problem. The community trusts us, but we know that the gap in staffing limits what we can deliver effectively. Male Health Staff, Mion |
|  | In my view, staffing is not adequate. We are only a few CHOs covering a large area, so sometimes services are delayed. The distribution also feels uneven, with some zones having more staff while others struggle with just one CHO. The composition is also narrow, as we lack midwives and other cadres who are vital for maternal care. Male Health Staff, Mion |
|  | The staff numbers are too low to meet community needs. At our CHPS zone, we mostly have CHOs, but few nurses or midwives. This composition limits the range of services we can provide. Distribution also creates pressure, zones with higher populations often have the same staff as smaller ones, which makes workload heavier in busier areas. Male Health Staff, Mion |
|  | From the community perspective, the staffing does not match our health needs. We see only one or two health workers at the CHPS, and they are overstretched. Important services like delivery care are limited. Sometimes people travel to other facilities because the staff here are too few or not the right type. Male Health Staff, Mion |
|  | Staffing in our CHPS is somewhat balanced. Outreach is fairly regular, and patients do not wait. The presence of female staff makes a big difference for antenatal services. Still, the absence of midwives means women sometimes seek TBAs. Male CHO, Nanton |
|  | Women here feel more confident accessing services because they know female nurses are available. This encourages antenatal visits and immunizations. However, without midwives, we cannot handle deliveries properly. Staffing is better than in Karaga, but still not fully adequate for our growing population. Male CHO, Nanton |
|  | I find that the distribution of staff here makes services more regular compared to some districts. However, the shortage of midwives and specialized staff still restricts what we can do. The workload is more manageable, but there is still pressure during campaigns and emergencies. Male CHO, Nanton |
|  | I support outreach activities, and it is easier here because staffing is relatively better. But sometimes we still fall short, especially for emergency cases where midwives are needed. The community trusts the CHPS compound more than in other places, but we are not yet where we should be. Female CHO, Nanton |
|  | On of my work involves mobilizing mothers for child health services. The presence of female nurses has made this easier because women respond better to them. Still, many women worry about delivering at the facility since midwives are not available. This remains a serious concern. Female CHO, Nanton |
|  | The services here are more consistent. Outreach is done regularly, and waiting times are shorter. But the shortage of midwives still forces women to depend on TBAs for delivery. This makes our work incomplete. Male Health Staff, Nanton |
|  | I work with record keeping, and with more staff available, it is easier to manage. However, we still face challenges when campaigns come up, because the workload increases drastically. The absence of permanent specialized staff like nutrition officers makes it difficult to expand our services. Male Health Staff, Nanton |
|  | Nanton is better staffed than some districts, but the gap is still clear. During emergencies, we often struggle because we lack midwives and specialized staff. The community notices this and keeps asking why those services are missing. Male Health Staff, Nanton |
|  | Women are more confident seeking care here than in other districts because female nurses are present. However, they still avoid delivering here due to the absence of midwives. This continues to weaken the trust in our maternal health services. Male Health Staff, Nanton |
|  | The presence of more CHOs here has reduced waiting times, and outreach is better organized. But without specialized staff, we cannot provide full services. The community feels the gap, especially when it comes to nutrition and delivery services. Female Health Staff, Nanton |
|  | The staffing situation in Nanton is still inadequate. Most CHPS zones rely on one or two CHOs, which makes it hard to cover all outreach and facility-based services. The composition is also limited, as we lack midwives and supporting cadres like disease control officers. When we have more female nurses, the women in the community are encouraged to go for antenatal care and delivery at the CHPS compound. Distribution is not balanced some zones have slightly better staffing, but others remain critically understaffed, affecting service delivery and community trust. Female Health Staff, Nanton |
|  | The adequacy of staff in our CHPS zones is low. Most compounds have only one CHO, making it difficult to deliver quality services. The composition is also skewed, with very few midwives or female health workers to handle maternal needs. Distribution is uneven, as some CHPS zones are staffed while others remain vacant, creating service gaps. Female Health Staff, Nanton |
|  | Staffing is not adequate for the workload. Often, a single CHO covers large communities, which affects outreach activities. The composition is limited there are no laboratory or nutrition staff, and female representation among staff is poor. Distribution is unfair, since remote CHPS zones are often left with only volunteers, while central zones receive more trained personnel. Female Health Staff, Nanton |
|  | From my experience, the staff distribution in Gushegu CHPS zones is not balanced. Some compounds have two or more workers, while others have none. The composition is weak, as we lack critical cadres like midwives. Adequacy is a problem across all zones, as demand for services far exceeds the available staff, leading to frustration among both workers and communities. Female Health Staff, Nanton |
|  | Staffing in Yendi is relatively better. This means we can organize outreach regularly and provide services without too many delays. However, the shortage of midwives still limits safe deliveries. Specialized staff occasionally join us, like nutrition or disease control officers, but they are not permanent. This weakens continuity of services. Male CHO, Yendi |
|  | Women in this district feel comfortable because female staff are available, and this encourages maternal and child health visits. With more CHOs, we are able to divide tasks better, but the absence of permanent midwives remains a gap. Pregnant women still prefer TBAs when they fear complications. If midwives were stationed here, service delivery would be much stronger. Female CHO, Yendi |
|  | I see that our staffing is adequate compared to some places like Gushegu, but the workload is still heavy. Outreach is regular, but emergencies are always a challenge because we lack midwives. Patients trust us, but when we refer them to distant hospitals for delivery, it reduces their confidence in the CHPS system. Female CHO, Yendi |
|  | I help with mobilization and education. With more CHOs here, community engagement is better organized. But during campaigns, even 15 staff are not enough for the population size. The shortage of midwives is a serious problem. Some women hesitate to attend the compound when they know they may still be referred elsewhere for delivery. Female CHO, Yendi |
|  | My work involves record-keeping and health education. Staffing is fairly balanced here, and patients experience fewer delays. But nutrition services are inconsistent since specialized staff are not stationed permanently. The community often asks about this, and it shows that numbers alone are not enough; diversity in staff matters too. Female CHO, Yendi |
|  | I have seen that having more staff makes service delivery smoother, but without midwives, our efforts are incomplete. Many women still rely on TBAs for delivery. This undermines the progress we have made in antenatal and child health services. More midwives are the most urgent need. Male Health Staff, Yendi |
|  | Women in the community trust the CHPS compound because they know female staff are available. This helps with antenatal care and immunization. But safe deliveries are still limited, and women often have to travel or use TBAs. For a district with 15 staff, the absence of midwives is still the biggest weakness. Male Health Staff, Yendi |
|  | Outreach services are more consistent here than in Gushegu or Karaga. But even with 15 staff, we struggle when the population demand is high. Specialized staff visit sometimes, but without their permanent presence, we cannot fully meet community health needs. Male Health Staff, Yendi |
|  | I manage records and follow-up visits. The workload is heavy, but at least we have enough staff to share responsibilities. Still, without midwives, women remain hesitant to deliver here. The presence of female nurses has helped antenatal care, but delivery remains a gap. Male Health Staff, Yendi |
|  | More staff here means patients wait less compared to other districts. Outreach is also more regular. But the absence of midwives is the most critical issue. We often hear community complaints that the CHPS compound cannot be trusted for delivery, even with many staff present. Male Health Staff, Yendi |
|  | My work is mainly health education, and I see that women respond positively to female staff. This has increased antenatal visits. But without midwives, many still depend on TBAs. We are working hard, but the community still sees a major problem in delivery services. Female Health Staff, Yendi |
|  | Staffing here is much better than in Karaga or Gushegu, but we still have challenges. Nutrition services are weak because staff are not permanently assigned. Delivery services remain poor without midwives. People appreciate the availability of services, but they still question why these key staff categories are missing. Female Health Staff, Yendi |
|  | The distribution of staff here is more balanced, which helps service delivery. But the absence of midwives means women still face risks. I think the community has confidence in us, but the trust is not complete because we cannot manage all maternal health cases. Female Health Staff, Yendi |
|  | I assist with logistics and mobilization. The CHOs are more here than in other districts, which makes it easier to handle large crowds. But the lack of permanent specialized staff and midwives still restricts us. The community expects more, and we are not able to deliver everything. Female Health Staff, Yendi |
|  | I feel that our district is better staffed. Outreach is more frequent, and waiting times are less. But the gap in delivery services due to lack of midwives is still serious. The community keeps asking when midwives will be posted here permanently. Female Health Staff, Yendi |
|  | Staffing in Karaga is very challenging. Even with 15 workers, the population is too large, and services are overstretched. Outreach is rare, and waiting times are long. The shortage of midwives is especially serious, forcing many women to rely on TBAs. Patients lose confidence when referred to distant hospitals. Female CHO, Karaga |
|  | Women in Karaga often complain about the absence of female midwives. They appreciate my presence for antenatal and child health services, but they are not confident about delivery here. With limited staff, outreach is not regular, and we often focus only on emergencies. This affects the community’s trust. Female CHO, Karaga |
|  | The workload here is extreme. Even though we have 15 staff, it does not feel enough because of the size of the district. Long waiting times are common, and patients are often frustrated. The absence of midwives makes maternal services weak, and TBAs remain influential. Female CHO, Karaga |
|  | I help with mobilization, but patients often complain that services are too slow. This is because there are too few staff compared to the population. Outreach is minimal, and when staff are exhausted, services stop. The absence of midwives is the main issue, as it forces women to seek deliveries elsewhere. Female CHO, Karaga |
|  | My work with records is difficult because the facility is often overcrowded. The few staff cannot attend to everyone, and patients sometimes leave without care. Women are particularly affected because there is no midwife to attend to them. This weakens confidence in our health services. Female CHO, Karaga |
|  | I assist CHOs with patient flow. the shortage of staff is clear. Outreach is very rare, and even at the compound, waiting times are unbearable. Without midwives, women prefer TBAs. This puts many lives at risk. Male Health Staff, Karaga |
|  | The population is large, but the staff are too few. Services are often delayed, and women are the ones who suffer the most. Many women are discouraged from seeking facility-based delivery because midwives are not available. This is our biggest challenge. Male Health Staff, Karaga |
|  | Outreach here is very limited because there are not enough staff to leave the compound. Women and children in remote areas are left out of services. Even at the compound, services are slow. The absence of midwives is a serious problem. Male Health Staff, Karaga |
|  | My role is to support health education, but even with this, the shortage of staff limits impact. People complain about long waits, and many do not return for follow-up. The community does not fully trust the compound because of these issues. Male Health Staff, Karaga |
|  | The staff composition is very poor here. No midwives, few female staff, and an overworked team. This makes it hard for women to seek services. Even antenatal care is weak because staff are stretched too thin. Female Health Staff, Karaga |
|  | I help with administrative work. Many patients leave without care because of delays. The shortage of staff, especially midwives, is the most serious challenge here. Without them, maternal and child health services are incomplete. Female Health Staff, Karaga |
|  | The shortage of specialized staff affects services. Nutrition and disease control are weak because the officers are not stationed here. The absence of midwives also makes delivery services very poor. This limits trust in the facility. Female Health Staff, Karaga |
|  | Our services are inconsistent because staff are too few. Outreach is rare, and even within the facility, long queues discourage people. Women are especially affected, as there are no midwives to attend to them. Famale Health Staff, Karaga |
|  | I help coordinate patient flow. the workload is extreme. Women do not feel confident using the facility for deliveries because no midwives are available. Patients often complain that the CHPS compound is not reliable. Female Health Staff, Karaga |
|  | I can say staffing here is relatively better. we can divide responsibilities and make outreach more consistent. However, we still lack midwives, which weakens delivery services. Specialized staff sometimes assist us, but without permanent postings, the impact is limited. Male Health Staff, Sagnarigu |
|  | Women in this community feel more encouraged because there are female nurses present. This helps with antenatal and immunization services. Still, the absence of midwives means deliveries are weak. We also need more specialized staff to make services broader and more reliable. Male Health Staff, Sagnarigu |
|  | With 15 staff, we are able to manage daily services, but the workload is still high. Patients benefit from shorter waiting times, but the absence of midwives makes maternal services incomplete. The community keeps asking when midwives will be assigned. Male Health Staff, Sagnarigu |
|  | I assist with mobilization and outreach. Staffing here is better than in Gushegu, but still not enough. Without midwives, deliveries remain unsafe. Specialized staff are not always available, and this reduces the scope of services we can provide. Male Health Staff, Sagnarigu |
|  | I manage records and patient follow-up. Services are smoother here than in Karaga, but the absence of midwives is still a major weakness. Women rely on TBAs for deliveries, and this undermines the progress we make in antenatal care. Male Health Staff, Sagnarigu |
|  | I have noticed that the presence of female staff encourages women to attend antenatal and immunization sessions. But without midwives, women still depend on TBAs. The workload here is manageable but not fully adequate. Male Health Staff, Sagnarigu |
|  | My work in health education shows that women trust the CHPS compound more when female staff are available. However, they still complain about the lack of midwives. Specialized staff visit, but without permanent presence, services are incomplete. Male Health Staff, Sagnarigu |
|  | Outreach is better here than in other districts, but still limited. Staffing numbers are higher, but without midwives, delivery services remain weak. The community feels this gap strongly. Female Health Staff, Sagnarigu |
|  | I help manage administrative work. Staffing is better here, but the absence of midwives and specialized staff limits our scope. Women do not fully trust delivery services without midwives. Female Health Staff, Sagnarigu |
|  | Patients here wait less compared to Karaga, but services are still not fully adequate. Without midwives, women feel unsafe delivering at the facility. Specialized staff are needed to improve quality. Female Health Staff, Sagnarigu |
|  | My role is in mobilization, and women respond positively because female staff are available. But without midwives, maternal services are still weak. The community feels this problem. Female Health Staff, Sagnarigu |
|  | Staffing is more balanced here, but still inadequate for the population. Outreach is better, but delivery services are poor due to lack of midwives. Specialized staff are also missing. Female Health Staff, Sagnarigu |
|  | I manage follow-up services. Women appreciate female staff, but without midwives, delivery is weak. People still depend on TBAs. The presence of specialized staff would make our services stronger. Female Health Staff, Sagnarigu |
|  | In Sagnarigu, staff numbers are relatively better compared to some neighboring districts, but the distribution is uneven. A few CHPS compounds have more than one CHO, while others rely on only one staff to handle all services. The composition is also narrow, with very few midwives or specialized health staff. This limits the range of services, especially maternal care. Female Health Staff, Sagnarigu |
|  | The staffing here is more encouraging than in some districts, particularly because we have more female nurses. Women in the community feel comfortable seeking antenatal and child health services from us. However, the overall adequacy is still a challenge. Outreach work suffers because staff are concentrated in certain zones, leaving other areas underserved. The shortage of midwives continues to be a big concern. Female Health Staff, Sagnarigu |
| How do you perceive the functionality of the CHPS in terms of service delivery, staffing, equity, and inclusiveness? | The CHPS compound is functional because staff and volunteers are always present. However, equipment shortages, especially for deliveries, weaken service delivery. Many women are referred to hospitals, which discourages them. Despite these challenges, trust has grown because people know health personnel are available at all times. Male CHO, Gushegu |
|  | Functionality is high, but equity gaps exist. Not all communities are reached equally because outreach depends heavily on volunteers. Since most volunteers are men, women sometimes hesitate to approach them for family planning discussions. The lack of female volunteers limits maternal service uptake. Male CHO, Gushegu |
|  | CHMCs and CHVs are active, and this supports strong functionality. However, basic diagnostic equipment is lacking. We rely on referrals for even simple cases, which affects the perception of quality. Still, the CHPS compound remains the first point of care for most households. Male CHO, Gushegu |
|  | The compound is functional, but supply of drugs is inconsistent. Mothers sometimes come for services but are told to buy medicines outside. This reduces trust. Women prefer female volunteers, but we have very few of them. This affects family planning and ANC discussions. Male CHO, Gushegu |
|  | Functionality is visible because staff and community structures work well together. However, persons with disabilities are not included in volunteer groups. They are treated more as clients than partners. This exclusion reduces the equity aspect of CHPS delivery. Female CHO, Gushegu |
|  | I feel services are strong, but the lack of specialized equipment is a gap. People still rely on the CHPS compound first because they trust the staff and volunteers. Equity problems are still there because youth and women are rarely selected for volunteer positions. Female Health Staff, Gushegu |
|  | CHPS compounds in this district function as intended, Only a few groups dominate volunteer positions. Young people complain they are not invited, and women struggle with household roles, so they remain underrepresented. Female Health Staff, Gushegu |
|  | Well service delivery is generally strong, and communities see the CHPS compound as reliable. But inequities in staffing and equipment availability are evident. Pregnant women in remote areas struggle to access complete services without midwives or diagnostic tools. Female Health Staff, Gushegu |
|  | Functionality is not the problem; resources are. Services run daily, but we lack equipment for basic diagnosis. Without delivery tools, maternal health remains compromised. The CHPS system is trusted, but people know its limits. Female Health Staff, Gushegu |
|  | The compound works, but women are less engaged in volunteer groups. Cultural expectations and household responsibilities prevent them from taking leadership. This makes women’s health issues less visible in outreach services. Female Health Staff, Gushegu |
|  | the CHPS compounds is functional, but service delivery suffers due to inadequate staff. We often work with only two people per zone, which makes outreach difficult. Equity is also an issue because remote communities wait longer for services. Inclusiveness is improving, but the lack of female staff in some zones limits maternal and child health care. Female Health Staff, Gushegu |
|  | The CHPS compounds provide essential services and are trusted by the community, but staffing is a real problem. We are too few to handle the workload, and without midwives, many women still rely on TBAs. women in remote areas cannot access the same level of care. Youth and minority voices are rarely involved in planning. Female Health Staff, Gushegu |
|  | From the community side, CHPS compounds are useful, but we see problems. Some compounds are always active like here, while others lack enough staff, so service is inconsistent. Women complain that without female nurses, they feel uncomfortable discussing maternal health. We also notice that people with disabilities are not included in activities. The system works, but not equally for everyone. Female Health Staff, Gushegu |
|  | Functionality is high, but equity issues persist. We try to involve everyone, but often the same people are selected as volunteers, and young people or minority groups are left out. This undermines inclusiveness, though service delivery is consistent with available staff. Male CHO, Mion |
|  | The CHPS compound is reliable, and people use it first. But, supply of drugs is inconsistent. Without medicines, women lose confidence. In addition, most volunteers are men, which discourages women from discussing sensitive health needs like family planning. Male CHO, Mion |
|  | Service delivery is active, and the CHPS compound fulfills its role. However, lack of diagnostic tools makes it difficult to handle even minor emergencies. Patients trust us but question why we always refer them out. Male CHO, Mion |
|  | Women trust the CHPS compound for antenatal and immunization. But equity gaps are clear: there are very few female volunteers. If more were recruited, mothers would feel safer discussing family planning. Equipment shortages also weaken our performance. Male CHO, Mion |
|  | I see high functionality in service delivery. But inclusiveness is weak. Youth and disabled persons are not represented in CHMCs or volunteer groups. They are treated as clients only, not as active participants. This limits equity. Male CHO, Mion |
|  | When women are part of the volunteer team, it encourages more mothers to attend ANC and talk about family planning. But in our CHPS, there are very few women volunteers. The compound itself is functional. Female CHO, Mion |
|  | Functionality is high, but supply issues reduce service quality. Essential drugs are often unavailable. People come first to CHPS, but they complain when asked to buy medicines outside. This erodes trust despite our efforts. Female CHO, Mion |
|  | Community trust is strong, and services are available. The same families are chosen for leadership, leaving out youth and minority groups. This exclusion weakens representation in CHMCs. Female CHO, Mion |
|  | Functionality is supported by volunteers, but most are men. Women hesitate to approach them on maternal issues. If more women were involved, services would be more equitable. Male Health Staff, Mion |
|  | The CHPS system works in terms of service delivery. However, marginalized groups, including youth and persons with disabilities, are excluded from volunteer roles. This limits inclusivity in the CHPS approach. Male Health Staff, Mion |
|  | The CHPS function fairly well, especially in providing maternal and child health services. However, staffing is inadequate. With only a few CHOs per zone, we cannot reach all communities regularly. Closer communities get more attention than distant ones and women and youth are rarely part of decision-making processes. Male Health Staff, Mion |
|  | We try our best with the resources available, but the functionality of CHPS is constrained. Service delivery is consistent for immunization and antenatal care, but emergencies are a challenge due to staffing shortages. Equity is affected by poor transport and long distances. Inclusiveness is improving, though female volunteers are underrepresented, making it harder to reach women effectively. Male Health Staff, Mion |
|  | The CHPS compound is functioning, but service delivery is stretched. Staffing levels are too low for the workload, and we lack specialists like midwives and nutrition officers. Poorer households cannot always travel to the compounds. Minority groups and persons with disabilities are mostly left out of community health structures. Male Health Staff, Mion |
|  | From our side, CHPS is helpful because it is the first place we go when children are sick or for antenatal care. But sometimes there are no drugs, and staff are few. Women feel it is easier when female nurses are available, but many compounds do not have them. Male Health Staff, Mion |
|  | Services are generally functional, with staff and volunteers always present. But drug supply is unreliable. Mothers come for ANC but are sometimes referred due to shortages. This undermines confidence despite the presence of staff. Male CHO, Nanton |
|  | Functionality is high, but female representation is weak. Women face cultural barriers that prevent them from volunteering. This affects outreach for maternal and child health. If women were more involved, uptake would increase. Male CHO, Nanton |
|  | The CHPS compound fulfills its role as the first point of care. But functionality is limited by lack of equipment. Diagnostic tools and delivery kits are absent. Patients still rely on CHPS for first contact, but confidence drops with referrals. Male Health Staff, Nanton |
|  | Services are functional, but there are equity concerns. Young people are rarely included in volunteer structures. They often say their contributions are undervalued. This discourages them from participating actively. Female CHO, Nanton |
|  | Functionality is not the issue equity is. Men dominate CHMCs and volunteer roles. Women feel excluded, especially in decision-making. This shapes how services are delivered and who feels comfortable accessing them. Female CHO, Nanton |
|  | The CHPS compound works well, but limited equipment constrains maternal care. Cultural barriers also prevent more women from joining volunteer groups. Without their presence, mothers hesitate to discuss family planning openly. Male Health Staff, Nanton |
|  | Services are functional, but supply shortages reduce trust. Essential medicines run out quickly. Patients still come here first but complain about the problems. Volunteers are reliable, but gender balance is missing. Male Health Staff, Nanton |
|  | Functionality is visible, but inclusivity is weak. Persons with disabilities are never considered for volunteer roles. They are seen as clients only, which undermines the spirit of equity in CHPS. Male Health Staff, Nanton |
|  | The compound is functional. Men dominate leadership roles, and women are left out. This limits how services respond to women’s specific needs. Male Health Staff, Nanton |
|  | You Know the CHPS zone functions well, but women and youth are not equally represented. If inclusivity improved, service delivery would be stronger. Female Health Staff, Nanton |
|  | Here the CHPS compound is functional but face serious challenges. Service delivery is regular for immunizations and antenatal services, but staffing is not enough to cover all outreach activities. Equity is affected because remote villages receive fewer visits than those close to the facility. Inclusiveness is weak, as women and youth are rarely part of planning discussions, leaving many voices unheard. Female Health Staff, Nanton |
|  | Functionality is high. Services run daily, and people trust us. However, lack of diagnostic equipment is a major problem. We cannot confirm cases easily and must refer. This reduces our perceived effectiveness. Male CHO, Yendi |
|  | When women are part of the volunteer team, it encourages more mothers talk about family planning. But in our district, women’s participation is still low. The CHPS system works, but equity is a concern. Female CHO, Yendi |
|  | The CHPS compound is reliable and is always the first point of care. But services are undermined by lack of delivery tools. Pregnant women lose confidence when told they must deliver elsewhere. Female CHO, Yendi |
|  | The CHPS compound is now the first point of care. People rely on it because the staff are always available and the volunteers help us reach the community. Functionality is strong. Female CHO, Yendi |
|  | Functionality is visible, but supply shortages affect maternal health services. Drugs and equipment are inconsistent. Mothers trust us but still complain about being referred to distant hospitals. Female CHO, Yendi |
|  | Functionality is good, but inclusivity is not. Volunteers are mostly men, and women feel shy to discuss family planning. If more women joined, uptake of maternal services would improve. Male Health Staff, Yendi |
|  | Services are available daily, but equity gaps exist. Young people are rarely selected for volunteer roles. Their contributions are undervalued, which makes them reluctant to participate in CHPS. Male Health Staff, Yendi |
|  | The CHPS compound functions. I want to say that persons with disabilities are excluded from volunteer groups. They are treated only as beneficiaries, not contributors. Male Health Staff, Yendi |
|  | Functionality is strong, but limited representation reduces inclusiveness. Male volunteers dominate, which discourages mothers from using certain services. Male Health Staff, Yendi |
|  | The CHPS compound works well, but drug shortages weaken trust. Patients complain that they cannot get medicines immediately. Volunteers help fill the gap, but equipment shortages remain. Male Health Staff, Yendi |
|  | The CHPS compound is functional and trusted. However, equity gaps exist because women and youth are rarely part of decision-making structures. Female Health Staff, Yendi |
|  | The CHPS in Yendi functions quite well because services like antenatal care, immunization, and malaria treatment are delivered consistently. However, staffing remains a problem; one or two CHOs cannot handle the workload across several communities. In terms of equity, poorer households still struggle to access timely services. Inclusiveness is limited, as community youth are rarely involved in decision-making. Female Health Staff, Yendi |
|  | Service delivery is strong, especially for maternal and child health, but staffing shortages is a problem. There are not enough nurses and volunteers to cover distant communities. Equity has improved slightly with community scorecard activities, but inclusiveness is weak. Women and young people are still underrepresented in CHMCs, so their perspectives are not fully integrated into planning. Female Health Staff, Yendi |
|  | The CHPS system is trusted here, but its functionality is not complete. Staffing numbers are too low, and support staff are stretched thin. Service delivery is regular, yet supply chain gaps undermine quality. Equity is not consistent: remote areas get fewer services, and inclusiveness is minimal because decision-making is dominated by a few influential leaders. Female Health Staff, Yendi |
|  | From my view, the CHPS is functional but struggles with resources and inclusiveness. Service delivery covers the basics, but referrals for emergencies are common due to lack of equipment. Staffing is inadequate, and female volunteers are especially few. This reduces women’s comfort in seeking maternal services. Inclusiveness is limited since persons with disabilities and minority groups are left out of community health structures. Female Health Staff, Yendi |
|  | Functionality is high, but the lack of equipment undermines quality. CHPS staff lack of basic diagnostic equipment and delivery tools undermining service quality, particularly in managing maternal health and referrals. Female CHO, Karaga |
|  | Services are generally functional. Women face cultural barriers that keep them out of CHMCs and volunteer groups. This weakens outreach for maternal services. Female CHO, Karaga |
|  | Functionality is good because staff are available. But lack of drugs and equipment weakens performance. Patients lose confidence when referred for simple cases. Female CHO, Karaga |
|  | The CHPS compound works but struggles with drug shortages. Functionality is not the issue but resources are. Staff are present, but they cannot perform effectively without tools. Female CHO, Karaga |
|  | The compound is functional, but women volunteers are absent. This makes it hard for mothers to seek sensitive services. Outreach is less effective without female representation. Female CHO, Karaga |
|  | Functionality is high, but inclusivity is weak. Minority groups are excluded from leadership and volunteer roles. This undermines the equity principle of CHPS. Male Health Staff, Karaga |
|  | Services run daily, and the CHPS system is trusted. But equity gaps persist because young people are rarely included in CHMCs. Their voices are missing. Male Health Staff, Karaga |
|  | Functionality is clear, but the absence of delivery tools weakens maternal care. Women still rely on TBAs because they cannot deliver safely at CHPS. Male Health Staff, Karaga |
|  | The compound is functional, but persons with disabilities are not included in volunteer groups. They are seen only as clients. This reduces inclusivity. Male Health Staff, Karaga |
|  | Functionality is good, but equity gaps exist. Men dominate volunteer teams, shaping how services are delivered. Women’s health needs are less prioritized. Female Health Staff, Karaga |
|  | The compound is reliable, but supply shortages affect maternal health services. Without basic tools, quality is undermined. Female Health Staff, Karaga |
|  | Functionality is visible, but equity concerns remain. Cultural barriers keep women out of leadership. This limits responsiveness to maternal health needs. Female Health Staff, Karaga |
|  | The CHPS system is functional and trusted, but minority and youth groups are excluded from volunteer teams. This affects inclusivity. Female Health Staff, Karaga |
|  | Services are reliable, but without equipment, quality remains low. Staff presence alone does not guarantee effective delivery. Female Health Staff, Karaga |
|  | Functionality is high here. Women and youth are left out of CHMC roles, and services reflect this imbalance. Female Health Staff, Karaga |
|  | Functionality is high, and the CHPS system is trusted. However, women are underrepresented in CHMCs. Cultural barriers and household roles limit their participation. Male Health Staff, Sagnarigu |
|  | The CHPS compound is now the first point of care. People rely on it because the staff are always available and the volunteers help us reach the community. Functionality is strong, but equity issues remain in representation. Male Health Staff, Sagnarigu |
|  | The CHPS compound functions well. But inconsistent drug supply affects community trust. Patients come first here, but they expect more. Male Health Staff, Sagnarigu |
|  | Service delivery in this CHPS is fairly functional, but the workload is high due to limited staff. We cover many communities with few staff. Equity is not always achieved, as remote villages get fewer visits. Inclusiveness also remains weak, especially for women and youth in community structures. Decision-making is often led by older men, leaving some groups underrepresented. Male Health Staff, Sagnarigu |
|  | The CHPS compounds are active and functional, especially in maternal and child health. However, staffing composition is inadequate; there are too few midwives and female staff to meet women’s needs. Equity in access is improving, but inclusiveness is limited. Women’s voices are still not well heard in CHMCs, and cultural barriers restrict their participation in health planning. Male Health Staff, Sagnarigu |
|  | CHPS functionality is visible, but service delivery faces many challenges. Staff numbers are insufficient, and volunteers are not well-trained. Equity is inconsistent; some groups, like persons with disabilities, are not reached equally. Inclusiveness in leadership roles is weak because community representation is narrow. Without involving women and marginalized groups, the full potential of CHPS is not realized. Male Health Staff, Sagnarigu |
|  | The CHPS system is trusted, and people come for services regularly. But staffing levels remain a major challenge. There are not enough health workers to manage the demand. Equity suffers because poorer and remote households face barriers to care. Also, women and young people are rarely given leadership roles within community health committees. Male Health Staff, Sagnarigu |
|  | Functionality is strong, but equipment shortages weaken quality. Even though staff are available, without diagnostic tools, our capacity is limited. Female Health Staff, Sagnarigu |
|  | Services are reliable and trusted. women are not well represented among volunteers. Mothers often ask why female volunteers are so few. Female Health Staff, Sagnarigu |
|  | Functionality is visible, but inclusivity is weak. Youth and minority groups are rarely included in volunteer groups. This reduces community representation. Female Health Staff, Sagnarigu |
|  | The CHPS compound works, but persons with disabilities are left out of leadership roles. They are clients only. Female Health Staff, Sagnarigu |
|  | Functionality is strong, but supply shortages undermine services. Patients still prefer CHPS as the first point of care, but they know its limitations. Female Health Staff, Sagnarigu |
|  | Services are consistent, and people rely on CHPS. But cultural norms keep women from leadership roles. This reduces inclusivity in decision-making. Female Health Staff, Sagnarigu |
|  | The CHPS system is functional and trusted. Yet, equity gaps exist due to limited participation of women and youth. This must change. Female Health Staff, Sagnarigu |
|  | The CHPS compound functions as expected, but without equipment, quality suffers. The system is reliable, but incomplete. Female Health Staff, Sagnarigu |
| How do you perceive the adequacy and impact of training on CSC and GESI-responsive CHAPs? | All CHOs in Gushegu were trained together on CSC and GESI-responsive CHAPs, which created a strong sense of capacity. Training emphasized community feedback collection and planning inclusively. With full coverage, staff can jointly apply CSC tools during meetings. Respondents credited strong district leadership and NGO support for ensuring all were trained, which has strengthened planning, accountability, and equitable service delivery across the CHPS zones. Male CHO, Gushegu |
|  | The CSC and GESI training sessions were highly impactful. For me, learning how to integrate women, youth, and persons with disabilities into community health planning has improved trust in our services. Since everyone received training, we share responsibilities equally, unlike districts with partial coverage. Challenges remain with resources, but our ability to plan inclusively and use CSC collectively is a strength that has made service delivery more accountable. Male CHO, Gushegu |
|  | Being trained on both CSC and GESI tools has enhanced how we engage with communities. We now collect feedback systematically and ensure diverse groups contribute to planning. Compared to other districts, Gushegu’s approach stands out because the training reached every CHO and staff member. This avoids reliance on only a few trained individuals. NGOs supported this effort, and strong district leadership ensured sustainability of what we learned. Male CHO, Gushegu |
|  | The training gave me confidence in addressing community concerns directly. Before, CHMC meetings lacked structure, but now, CSC tools guide discussions. We also learned to consider GESI dimensions, especially involving youth and women. This inclusive planning is a big improvement. Because training was universal here, we work collectively, unlike colleagues in other districts who depend on a handful of trained CHOs. Our district’s experience is a model worth replicating. Male CHO, Gushegu |
|  | We all attended CSC and GESI training sessions, which changed how we operate. Collecting structured feedback from households is easier now. Importantly, GESI training showed us the value of including women and vulnerable groups in our CHAPs. Many communities feel their concerns are finally heard. I know other districts face knowledge gaps, but Gushegu avoided that by ensuring total staff participation, supported by NGOs and strong leadership. Female CHO, Gushegu |
|  | Full training on CSC and GESI-responsive CHAPs means I feel equally equipped as my CHO colleagues. We can identify accountability gaps and address equity concerns. This avoids depending only on CHOs for community engagement. Other districts have not been so fortunate, but Gushegu’s collective training ensures shared knowledge and accountability. It is easier to sustain progress when everyone understands the principles of inclusion and social accountability. Female Health Staff, Gushegu |
|  | The CSC and GESI training made our work easier by teaching us how to record and address complaints systematically. We also learned to involve women and vulnerable groups in planning. Because the training was comprehensive, all CHPS staff are competent in applying these tools. This makes service delivery more transparent. It is clear that our district’s success comes from coordinated leadership and external NGO support that other districts lack. Female Health Staff, Gushegu |
|  | Having attended CSC and GESI sessions, I see how inclusive planning improves trust. Now, community voices are better represented, particularly those of women and youth. Unlike in districts with limited training, we all understand the approach, so service delivery does not depend on a few people. This has reduced conflict in decision-making and improved collaboration. Gushegu has benefitted from good leadership and NGO support to achieve this coverage. Female Health Staff, Gushegu |
|  | After CSC training, I became more confident in facilitating community scorecard sessions. The GESI training added depth by showing how to involve marginalized groups in discussions. The fact that every staff member was trained has created collective ownership. It prevents the “knowledge bottlenecks” that weaken other districts. Our leadership pushed for complete participation, and the outcome is more inclusive, accountable CHPS planning processes. Female Health Staff, Gushegu |
|  | Attending both CSC and GESI-responsive CHAPs training allowed me to improve how I engage women in maternal health discussions. The training also emphasized equity, encouraging us to include youth and disabled persons. Because everyone was trained together, our teamwork is stronger. We now plan inclusively, unlike in districts with partial coverage where implementation depends on a few trained staff. Female Health Staff, Gushegu |
|  | The CSC training made me better at facilitating structured accountability sessions with communities. The GESI training taught us to consider social inclusion during CHAP development. Since all CHPS staff were trained, we apply this knowledge as a group, not individually. Our district is fortunate to have had leadership and NGO backing to support this comprehensive approach. Male Health Staff, Gushegu |
|  | With CSC training, I learned to gather and document community feedback systematically. GESI training encouraged us to recognize the voices of vulnerable populations when drafting CHAPs. Because the training reached everyone, knowledge sharing is not limited. This inclusive capacity building has been transformative. Other districts lack this coverage, but Gushegu has demonstrated what collective training can achieve. Male Health Staff, Gushegu |
|  | Training on CSC gave us practical tools to assess service delivery and improve accountability. GESI training highlighted gender and equity gaps, ensuring women and youth are included in planning. Our district benefited from total training coverage, so there are no gaps in capacity. Staff work as a team, and no one is left behind. This prevents the common bottlenecks seen in districts with partial or no training. Male Health Staff, Gushegu |
|  | In Mion, training coverage was partial. About half of us received CSC training, while others were left out. This caused challenges, as knowledge was concentrated in a few staff. I benefited from learning how to collect and analyze feedback from communities, but my colleagues who missed training struggle to apply CSC. On GESI, we had some exposure, which helped us see the value of including women and youth, though coverage was uneven. Male CHO, Mion |
|  | I was among the few in Mion trained on CSC. It was very useful, I learned about scorecards and accountability processes. But many of my colleagues didn’t attend. Only a handful of us can implement CSC properly. On GESI, the training we received helped us think about inclusivity, but again, only part of the staff had exposure. This makes sustaining the practice difficult, as knowledge is unevenly distributed. Male CHO, Mion |
|  | Here in Mion, only some staff got trained on CSC. I was not included, so I depend on colleagues for updates. This creates gaps because we cannot all apply the tool consistently. On GESI, training reached a few of us, but not all. As a result, inclusion of women and vulnerable groups in planning is inconsistent. This selective approach makes the district’s overall CSC and GESI practice weak compared to where full coverage was done. Male CHO, Mion |
|  | the CSC training was helpful for those who attended, but not everyone did. I was trained, so I know how to use the scorecards. However, in meetings, I see others struggling because they were excluded. The same applies to GESI training it opened my eyes to equity issues, but many colleagues didn’t get that knowledge. This makes teamwork harder because implementation often relies on one or two people instead of the whole staff. Male CHO, Mion |
|  | Partial training created uneven capacity in Mion. I learned how CSC works and why it’s important for accountability, but my colleagues who missed the training cannot contribute. This leads to gaps when we try to do joint activities. On GESI, only some staff attended. While I now understand how to involve women and youth in CHAPs, the team as a whole cannot fully apply this without refresher or broader coverage. Male CHO, Mion |
|  | I can say the training on CSC in Mion was useful, but the problem is that only a portion of us got it. I learned how to document community feedback, but during planning, we depend heavily on those few who were trained. The GESI session taught me the importance of inclusion, yet many others did not benefit. This imbalance reduces the effectiveness of our work and creates bottlenecks in implementation. Female CHO, Mion |
|  | In our district, CSC training covered some staff but left others behind. I was not trained, so I often feel lost when CSC discussions come up. The GESI training was also selective, which means planning is not consistent. We end up relying on colleagues who were trained, and that slows progress. It creates dependency, rather than collective knowledge. If all of us had been trained, we would perform better as a team. Female CHO, Mion |
|  | I received CSC training and found it valuable. It showed me how to collect feedback and use it in service planning. But not everyone in Mion got the same chance. This has led to uneven skills among the staff. GESI training was also not given to everyone, so inclusion of women and vulnerable groups varies. Without broader training coverage, we struggle to institutionalize CSC and GESI practices across the CHPS zones. Female CHO, Mion |
|  | Our challenge in Mion is inconsistency. I learned from CSC training that accountability depends on structured feedback. But since many staff were not trained, they cannot support the process. On GESI, only a few of us were exposed to the training. This makes it hard to sustain, because when those trained are absent, the knowledge gap is very clear. This uneven training coverage weakens our district’s overall performance. Male Health Staff, Mion |
|  | I participated in CSC training and saw its value. It improved my understanding of how to make communities part of planning. But only some of us were invited, which means knowledge was not shared equally. The same happened with GESI training very few were covered. As a result, implementation often depends on one or two individuals instead of being a collective effort. This is why we face what I call ‘knowledge bottlenecks’ in Mion. Male Health Staff, Mion |
|  | The training we had in Mion was not enough to reach everyone. Some staff, including myself, missed CSC training. This limits our ability to implement it fully. For GESI, only selected staff were trained, so inclusivity is not always practiced in CHAPs. The uneven training weakens consistency across the team. Unlike Gushegu, where everyone was trained, Mion still depends on a small number of trained individuals to lead the process. Male Health Staff, Mion |
|  | In Mion, I noticed that partial training caused dependency. I was trained on CSC, but my colleagues often rely on me because they didn’t attend. This makes implementation slower and less effective. GESI training was the same only a few got exposure. Although I now understand how to involve marginalized groups in planning, the team cannot do this consistently. Wider training coverage would have avoided these gaps. As it stands, we face challenges. Male Health Staff, Mion |
|  | Mion’s training was uneven. I learned about CSC and how to use scorecards, but many others missed it. This creates bottlenecks, since implementation depends on the trained few. On GESI, coverage was also limited, leaving many staff unaware of how to make planning inclusive. The district needs broader training to ensure collective practice. Otherwise, we will keep struggling with gaps and dependency on only a handful of trained workers. Female Health Staff, Mion |
|  | The CSC training gave me skills to improve accountability with communities, but in Mion not all staff were included. This unevenness means only a few of us can apply the method. On GESI, I learned about equity, but again, training didn’t cover everyone. As a result, planning still misses vulnerable voices in some cases. The partial approach has created what we call knowledge bottlenecks, where only one or two people can lead. Female Health Staff, Mion |
|  | In Nanton, none of us received CSC training. We are expected to apply accountability tools, but we simply lack the knowledge. It has been difficult to measure community feedback systematically. On GESI, there was also no training. This means our planning does not fully include women, youth, or vulnerable groups. Without training, we feel left behind compared to other districts, and it reduces our ability to respond to community needs effectively. Male CHO, Nanton |
|  | We never had CSC training in Nanton, and that creates big challenges. I know about it in theory, but I don’t know how to apply it. The same applies to GESI training, which was never provided here. We often plan activities without specific attention to gender or vulnerable groups because we lack the skills. This gap leaves community voices underrepresented and makes our work less inclusive than it should be. Male CHO, Nanton |
|  | In our district, no CSC training was done. This makes accountability difficult because we do not have the right approach to collect community feedback. We rely on informal discussions instead of structured tools. GESI training was also not conducted, so inclusion in our health plans is limited. Many times, women’s or youth perspectives are missing. This leaves us behind compared to other districts and weakens trust in our CHPS structures. Male CHO, Nanton |
|  | We never got training on CSC or GESI in Nanton. This makes it very hard for us to practice accountability and inclusivity. When communities ask about scorecards, we cannot respond properly. For GESI, I feel it is a missed opportunity, because as women, we see clearly how gender shapes access to services, but we lack tools to address it. Without training, we keep doing things the old way, which excludes many voices. Female CHO, Nanton |
|  | The challenge in Nanton is that there has been no CSC training at all. I have read about it but cannot apply it practically. It weakens our accountability to communities. On GESI, the absence of training means we don’t know how to integrate equity issues into CHAPs. This gap is serious because our planning tends to overlook women and youth. Without training, CHPS here cannot be as effective as in other districts. Female CHO, Nanton |
|  | Here in Nanton, the absence of CSC training means we don’t have structured tools to assess service delivery. We rely only on feedback during durbars, which is not systematic. On GESI, the situation is the same no training was given. As a result, our CHAPs often miss the needs of women, youth, and vulnerable groups. It feels like the district is left behind while others move forward with new knowledge and approaches. Male Health Staff, Nanton |
|  | No CSC training was delivered in Nanton, so when we are asked to show evidence of accountability, we don’t know how to proceed. It is frustrating. On GESI, no training was conducted either, meaning inclusivity is not part of our standard planning. This is a gap because women and youth are central to health services. Without training, our district remains dependent on outdated methods, and communities lose confidence in our structures. Male Health Staff, Nanton |
|  | In Nanton, we did not have any CSC training. This has made it hard for us to apply accountability frameworks. The same with GESI there was no training. I feel this exclusion weakens our capacity as health workers. We plan activities without full consideration of vulnerable groups. For me, this is disappointing because training could help us address these equity gaps, but without it, our CHPS compounds continue to operate in a limited way. Male Health Staff, Nanton |
|  | The lack of CSC training in Nanton is a serious challenge. We know the tool exists, but we cannot use it. This limits our ability to track service delivery quality. On GESI, there was also no training, so gender and social inclusion remain weak points in our planning. Communities often ask why their concerns are not fully reflected, and I believe the answer is clear we lack the knowledge and skills that training provides. Male Health Staff, Nanton |
|  | We feel disadvantaged in Nanton because no CSC training has been conducted. When we hear that other districts are using the tool, we realize how much we are missing. On GESI, there is also a gap no training at all. This means our CHAPs do not fully consider equity issues. Women, youth, and persons with disabilities are often left out unintentionally. I think training would change this, but we have none here. Female Health Staff, Nanton |
|  | None of us in Nanton were trained on CSC, so applying it is impossible. We end up using guesswork or informal meetings to gather community feedback. GESI training was also not done, and that makes inclusivity weak. The absence of these trainings has made our CHPS zones less effective than in places like Gushegu. We cannot compare because our knowledge is limited. This gap directly affects the quality of health services here. Female Health Staff, Nanton |
|  | The reality in Nanton is that there has been no training on CSC or GESI. This leaves us behind in accountability and equity. We hear about these concepts from colleagues in other districts, but here there is no practical knowledge. I believe this weakens both our planning and our ability to involve communities. Without training, CHPS functionality remains limited, and marginalized voices continue to be excluded from decision-making processes. Female Health Staff, Nanton |
|  | In Nanton, none of the staff has been trained on CSC. This creates a serious gap in our accountability work. For GESI, the same applies we have had no training. The effect is that CHAPs are developed without structured input from women, youth, or vulnerable groups. Our plans are therefore incomplete. It is frustrating to know that other districts benefit from such training while we are left without the tools to improve services. Female Health Staff, Nanton |
|  | I can say clearly that no CSC or GESI training was done in Nanton. This is why we often struggle to include community voices in CHAPs. Without CSC, we lack structured accountability. Without GESI, planning remains biased toward majority voices, overlooking women, youth, and minority groups. This is a big limitation for us as health staff. Training would change our capacity, but right now, we remain untrained and disadvantaged compared to other districts. Female Health Staff, Nanton |
|  | In Yendi, I was trained on CSC and it has improved how we gather community feedback. We now use structured tools instead of relying only on meetings. For GESI, the training helped us integrate women, youth, and vulnerable groups into CHAPs. The training opened our eyes to how to include women, youth, and vulnerable groups in our health plans. But without refresher training and support, it is hard to sustain. Also, not every staff member attended, so knowledge is uneven. Some colleagues depend on me to guide them. Refresher training would help sustain this progress and make it a collective practice. Male CHO, Yendi |
|  | The CSC training in Yendi was very practical. I can now document community concerns better and follow up with action. The GESI training was also useful, because it made me consider groups often left out, like women with disabilities. But not all staff were trained, especially midwives, which creates gaps. Sometimes only a few of us apply these tools, while others continue the old way. Wider coverage is needed for full impact. Female CHO, Yendi |
|  | I benefited from CSC training, and it completely changed how I view accountability. Communities now feel more confident to give us feedback. The GESI-responsive CHAPs training also helped us bring women and youth into the planning process. The problem is that not everyone got the training. For example, some enrolled nurses did not attend. This creates reliance on a few trained individuals rather than collective practice across the entire CHPS zone. Female CHO, Yendi |
|  | In Yendi, both CSC and GESI training were conducted. I found the GESI part especially eye-opening, because it emphasized including women and youth in planning. Before, we focused mainly on general health outcomes. Now, we try to be more inclusive. Still, the challenge is sustainability. We need refresher training and district-level support. Without that, the momentum will fade, and planning might slip back into ignoring marginalized voices. Female CHO, Yendi |
|  | “We received CSC training, and it improved our accountability. Communities feel their voices are heard because we use scorecards. The GESI training also shaped our planning—now we make deliberate space for women and youth. However, some staff were excluded, like midwives. That limits our ability to fully integrate equity, especially for maternal and child health. If all cadres had been trained, the impact would have been stronger and more consistent. Female CHO, Yendi |
|  | The trainings on CSC and GESI were transformative. I now understand better how to capture community views systematically and make sure equity is addressed in our health plans. Women’s groups in particular appreciate being consulted. The limitation is that only part of the staff got trained. Without refresher courses or broader participation, knowledge risks staying with a few individuals instead of spreading across all CHPS zones. Male Health Staff, Yendi |
|  | The CSC training gave me practical tools to engage communities. People feel included when they see their feedback recorded and discussed. The GESI training also highlighted the need to deliberately involve women and vulnerable groups. But challenges remain. Not every staff member participated, which creates gaps. Sometimes implementation depends on one or two trained individuals, making it fragile. Expanding coverage and refreshing training would help strengthen accountability and inclusivity. Male Health Staff, Yendi |
|  | I think Yendi is lucky because both CSC and GESI trainings were offered. I was part of both, and I feel more confident in planning now. For example, we intentionally ask women’s associations to give inputs into CHAPs. But not all my colleagues have this knowledge, so application is inconsistent. Midwives, who are central to maternal care, were left out. That weakens inclusivity. I hope future trainings will include every category of health staff. Male Health Staff, Yendi |
|  | The CSC training was very effective in teaching us accountability. We now apply scorecards to assess services. The GESI-responsive CHAPs training also opened our eyes to gender equity. I use these lessons daily. But coverage was limited. Some colleagues still do not know how to apply CSC. That creates bottlenecks when we are planning. It would help if all health staff, especially those working closely with mothers, received similar training. Male Health Staff, Yendi |
|  | In Yendi, both trainings helped improve our planning. The CSC component made it easier to involve communities systematically, and the GESI part ensured we include groups that were often left out. For me, this was a big improvement. However, coverage was uneven. Some colleagues had no exposure at all, which makes teamwork difficult. We end up carrying the burden of sharing knowledge informally. District support is needed for full implementation. Male Health Staff, Yendi |
|  | I took part in both trainings, and they have been very useful. CSC gave us tools for accountability, and GESI training helped us include marginalized groups in our health plans. Still, there is an issue of sustainability. Training was done once, and not everyone was reached. We need refresher sessions and wider coverage. Without that, only a few of us practice it properly, while others continue with business as usual. Female Health Staff, Yendi |
|  | The CSC and GESI trainings we had in Yendi were very important. Before, I didn’t think much about youth or disabled persons in health planning. Now, I try to bring those voices in. But the trainings did not cover everyone. Many staff, especially midwives, were excluded. This creates inequality even within our team. We need training to reach all categories of staff, otherwise inclusivity in CHAPs will remain incomplete. Female Health Staff, Yendi |
|  | Being trained on CSC has made accountability easier. Communities now trust us more, because we collect feedback in an organized way. GESI training also taught us to integrate equity into CHAPs. However, training coverage was partial. Only a few CHOs and nurses attended. Without district-level support to extend coverage and give refresher training, these tools risk being underutilized. Implementation depends too much on a handful of trained staff. Female Health Staff, Yendi |
|  | I was fortunate to attend both CSC and GESI trainings. They really shaped my perspective, especially about including women and vulnerable groups in health planning. Now, when we prepare CHAPs, I push for their voices to be heard. The issue is that many of my colleagues were not trained. This creates knowledge gaps, and some continue planning without these approaches. To make it sustainable, the district should prioritize refresher trainings for everyone. Female Health Staff, Yendi |
|  | Yendi has benefitted from CSC and GESI training, and I was part of both. The CSC scorecard system makes accountability more structured, while GESI emphasizes inclusivity. Still, not every staff member is trained, so coverage is incomplete. For example, some cadres like midwives and enrolled nurses did not participate. This leaves important service areas without equity-oriented planning. More resources are needed to cover everyone and make the training sustainable. Female Health Staff, Yendi |
|  | In Karaga, we did not receive any CSC training, so we still rely on community durbars and informal meetings to get feedback. There is no structured system like scorecards. As for GESI-responsive CHAPs, nothing was offered. This makes it difficult to plan inclusively. We often default to traditional ways of consultation, which exclude women and youth voices. It feels like we are being left behind compared to other districts. Female CHO, Karaga |
|  | We heard colleagues in other districts received CSC and GESI training, but here in Karaga nothing was organized. We have no tools for structured accountability, and CHAPs are prepared without much focus on inclusion. Women, youth, and people with disabilities rarely get considered. It is frustrating, because we see the benefits elsewhere but cannot apply them here. Without training, we cannot adapt our methods or improve equity in health planning. Female CHO, Karaga |
|  | In Karaga, the absence of training is a big gap. We rely on traditional engagement methods, which are not systematic. There is no CSC approach, and no awareness about GESI-responsive planning. As a result, our CHAPs are generic and fail to highlight the needs of vulnerable groups. This leaves us frustrated, because we want to do better but lack the knowledge and skills to implement modern accountability and equity approaches. Female CHO, Karaga |
|  | No CSC training has been provided in Karaga. We try to listen to communities, but it is very informal, and not everyone feels comfortable to speak up. Without GESI-responsive CHAPs training, women and youth perspectives are overlooked. I feel our district is disadvantaged compared to Yendi or Kintampo. We do not have the skills to capture marginalized voices systematically, so our health plans remain incomplete and less responsive. Female CHO, Karaga |
|  | The lack of CSC training here is clear. We have no scorecards or structured accountability tools. We just hold meetings, and whoever speaks is heard. This excludes many groups, especially women and young people. With no GESI training, we also do not know how to integrate gender or equity into CHAPs. It feels like our district was bypassed in capacity building. The result is weaker community trust and less inclusive planning. Female CHO, Karaga |
|  | We hear about the CSC, but we have never been trained. We are expected to use it, but we don’t know how. Karaga has not received any of the CSC or GESI trainings, and this is a serious gap. Our CHAPs are developed without systematic community input. Women’s groups are often not consulted, and people with disabilities are invisible in our plans. We rely on old methods that are not enough. Staff feel excluded from opportunities that colleagues in other districts benefitted from. This makes us less effective in delivering equitable services. Male Health Staff, Karaga |
|  | In Karaga, accountability is very weak because we never had CSC training. We lack the tools to collect feedback properly. For GESI-responsive CHAPs, there was also no training. So our plans are drafted in the old way, with limited participation. It is frustrating because we know there are better ways used elsewhere, but here we are left behind. This creates a big equity gap in how districts approach planning. Male Health Staff, Karaga |
|  | Without CSC training, community feedback remains unstructured. Some people talk, others stay silent, and nothing is documented. We also missed out on GESI training, so there is no deliberate inclusion of women, youth, or vulnerable groups. As a result, CHAPs are not gender-responsive. We feel like Karaga is excluded from development. This gap affects the quality of services and leaves staff frustrated, because we want to do more but lack skills. Male Health Staff, Karaga |
|  | We never had CSC training in Karaga. Accountability is therefore weak, and planning is not inclusive. With no GESI-responsive CHAPs training, there is no deliberate attempt to capture marginalized voices. We depend on chiefs and community leaders to speak for everyone, which is not fair. Other districts are advancing with new skills, but we are left out. This creates inequality in service delivery between districts, and it demoralizes staff. Male Health Staff, Karaga |
|  | In Karaga, we prepare CHAPs without CSC or GESI training. This means feedback collection is ad hoc and not structured. Women’s voices are often missing, because they do not attend community meetings in large numbers. Without gender-sensitive tools, inclusivity is weak. Staff like me feel frustrated because we know the gap is not our fault. Training would empower us, but we have been excluded from these important opportunities. Female Health Staff, Karaga |
|  | No CSC training means no systematic accountability here in Karaga. We cannot compare ourselves with districts like Yendi, where communities are actively involved. Similarly, without GESI-responsive training, gender and equity issues are neglected. CHAPs are produced, but they fail to address the needs of the most vulnerable. It feels unfair that our district has been left behind. Staff are willing to learn, but there are no opportunities provided. Female Health Staff, Karaga |
|  | I feel Karaga is missing out because we never had any CSC or GESI trainings. Our health plans are done in the usual way, with chiefs and elders consulted, but women and youth excluded. If we had training, we could make CHAPs more inclusive. Instead, the absence of capacity building has left us with weak accountability structures. It is frustrating to watch other districts progress while ours struggles with outdated methods. Female Health Staff, Karaga |
|  | The absence of CSC training has left Karaga behind. We do not use scorecards or systematic community monitoring. Planning is top-down, with little inclusion. Without GESI training, CHAPs lack sensitivity to gender or vulnerable groups. This limits the effectiveness of our work. Staff are demotivated because they see others advancing. We want to be trained, but no opportunities have come. It creates a sense of exclusion and inequity between districts. Female Health Staff, Karaga |
|  | Karaga staff were not included in CSC or GESI training. We therefore lack the tools to involve communities meaningfully. Our CHAPs often miss the needs of women, youth, and marginalized groups. The absence of training creates frustration because we want to improve but cannot. It feels like the district is forgotten. Without skills, accountability and inclusivity remain weak, and our planning cannot match the standards seen in trained districts. Female Health Staff, Karaga |
|  | Here in Karaga, no CSC or GESI training has been conducted. Our CHAPs are prepared using the old methods, with elders consulted but no structured way to hear from women or youth. We lack accountability tools and equity perspectives. This has left us disadvantaged compared to other districts. Staff feel excluded and unprepared. If we had training, we could do better, but without it, inclusivity and accountability remain weak. Female Health Staff, Karaga |
|  | In Sagnarigu, only a few of us were trained on CSC, and that has created knowledge bottlenecks. Those trained cannot cover all CHPS zones, and implementation depends on one or two staff. The absence of GESI-responsive CHAPs training means our health plans ignore women and vulnerable groups. We try to be accountable, but without gender-sensitive planning skills, inclusivity is weak. This leaves us frustrated and limited in practice. Male Health Staff, Sagnarigu |
|  | CSC training was given, but coverage was very small. I was not included, so I depend on colleagues to explain. That makes accountability inconsistent. Without GESI-responsive CHAPs training, our CHAPs are not designed to involve women, youth, or persons with disabilities systematically. As a result, equity gaps remain wide. Staff like me feel excluded, and the community feels their voices are missing in our health plans. Male Health Staff, Sagnarigu |
|  | Only a handful of CHOs in Sagnarigu were trained in CSC, and knowledge transfer is weak. Many of us still do not understand how to use scorecards. As for GESI training, we had none at all. This means our health plans miss the needs of women and marginalized groups. The lack of inclusion frustrates staff. We know the gaps exist, but without proper training, we cannot integrate equity into our planning. Male Health Staff, Sagnarigu |
|  | We never had GESI-responsive CHAPs training in Sagnarigu, and CSC training reached very few. I was excluded, and so were most nurses. The trained staff try to share knowledge, but it is not enough. Women and youth continue to be underrepresented in planning. As midwives, we are central to maternal health, yet excluded from training. This makes our CHAPs less inclusive and our accountability incomplete, even though we are committed to equity. Male Health Staff, Sagnarigu |
|  | CSC training covered only a small proportion of staff here in Sagnarigu. Those who attended cannot apply it everywhere. Without GESI training, inclusivity in CHAPs is missing. Health plans are written in the usual way, with little focus on equity. Women, youth, and minority groups are overlooked. This frustrates many of us because we see how accountability could improve, but the lack of training and resources keeps us behind. Male Health Staff, Sagnarigu |
|  | The CSC training in Sagnarigu was very limited. I was left out, and I depend on second-hand explanations from colleagues. Without GESI-responsive training, we are not taught how to plan inclusively. Our CHAPs fail to address equity issues. Women and vulnerable groups continue to be excluded in subtle ways. As CHOs, we want to improve, but being excluded from training reduces our ability to apply accountability and inclusivity in daily work. Male Health Staff, Sagnarigu |
|  | A few CSC trainings were done, but I was not selected. This has created knowledge gaps. The district depends on one or two staff to lead accountability, which is not sustainable. With no GESI training, gender and equity issues are ignored. Our CHAPs are therefore incomplete and fail to address community diversity. Staff feel frustrated, because we want to plan inclusively but are not equipped to do so without the right skills. Male Health Staff, Sagnarigu |
|  | In Sagnarigu, midwives like us were not included in CSC training, and none of us received GESI training. This leaves a big gap. We work with mothers every day, but we cannot integrate gender-sensitive planning into CHAPs. Our district talks about equity, but without training, it remains theory. The small CSC coverage creates bottlenecks where only a few people have knowledge. That weakens accountability and frustrates staff commitment to inclusivity. Female Health Staff, Sagnarigu |
|  | We work directly with mothers every day, but no one has trained us on CSC or GESI. It feels like these trainings are only for CHOs. CSC training was only for a few people in our district. We try to use scorecards where possible, but without wider training, accountability is weak. Since no GESI training was provided, our CHAPs remain gender-blind. Youth, women, and minority voices are often missing. This shows how unequal training distribution undermines planning. Staff like me want to make changes, but the lack of exposure prevents us from applying inclusive approaches. Female Health Staff, Sagnarigu |
|  | The challenge in Sagnarigu is that CSC training covered only a fraction of the staff. Most of us were left out. That creates bottlenecks because only one or two people know how to use CSC tools. With no GESI training, inclusivity is left aside. Planning often excludes women and youth. As a CHO, I feel we are working with incomplete tools. Accountability is discussed, but in practice, it is very weak due to poor training. Female Health Staff, Sagnarigu |
|  | I was not part of the small CSC training here in Sagnarigu, and that has left many of us unprepared. Knowledge does not spread easily. The absence of GESI-responsive CHAPs training further widens the gap, as our CHAPs do not consider gender or disability issues properly. The result is health plans that are less responsive to equity needs. Staff want to do better, but being excluded from training keeps us dependent and limited. Female Health Staff, Sagnarigu |
|  | The problem here is that CSC training reached very few. Most staff, including myself, were excluded. That leaves us unable to apply accountability in our daily work. With no GESI-responsive training, inclusivity is missing in CHAPs. Women and vulnerable groups are not systematically involved. It feels like our district is left behind compared to Gushegu or Yendi. Staff are frustrated because the training gaps reduce our effectiveness in planning. Female Health Staff, Sagnarigu |
|  | CSC training was introduced but only for a handful. This has created dependence on those trained. The majority of us do not know how to apply scorecards. Without GESI training, our CHAPs ignore key equity concerns. Women, youth, and persons with disabilities are not systematically included. The exclusion from training is discouraging because we want to learn. Our district remains behind others in accountability and inclusivity. Female Health Staff, Sagnarigu |
|  | In Sagnarigu, partial CSC training was done, but I was excluded. This means accountability work relies on a few trained colleagues. Without GESI-responsive CHAPs training, our health plans are not inclusive. Women’s voices are missing, and disability is rarely considered. Midwives and nurses are also left out of training, which reduces effectiveness. The lack of coverage has created frustration among staff, because we want to integrate equity but cannot. Female Health Staff, Sagnarigu |
|  | We only had partial CSC training, with very low coverage. Most staff are left out, so accountability remains weak. With no GESI-responsive CHAPs training, gender and equity are ignored. Our CHAPs are not inclusive, and women, youth, and minority groups are often excluded. Staff feel left behind compared to districts that had full training. Female Health Staff, Sagnarigu |
| How have Community Scorecard (CSC) recommendations influenced improvements in CHPS service delivery? | The CSC process helped us see gaps in our services. The community mobilized resources to renovate the facility, improve water and sanitation, and provide shade for patients. These actions boosted both staff motivation and patient trust. Even small improvements like building shade structures made a big difference. It showed us that when communities are engaged, they can directly support maternal and child health services and strengthen our delivery capacity. Male CHO, Gushegu |
|  | In Gushegu, the CSC created a platform where staff and the community could identify gaps together. This led to joint actions such as improving hygiene, repairing facility structures, and expanding outreach for maternal care. The participation of community members was important because it increased accountability. Service delivery has improved, especially antenatal visits and outreach sessions, because the community feels ownership. The partnership created by the CSC has helped sustain these changes. Male CHO, Gushegu |
|  | The CSC revealed many service delivery issues, especially with maternal and child health. The community acted quickly by supporting the repair of the facility and contributing to water improvements. These changes encouraged more mothers to attend antenatal care and immunizations. As staff, we feel supported because the community understands our challenges and works with us. Without their contribution, many of these service improvements would not have been possible or sustainable. Male CHO, Gushegu |
|  | The CSC made it clear what we were lacking, and the community took it upon itself to act. Even small changes like building shade structures made a difference for staff and patients. They assisted in renovating parts of the facility, provided clean water, and supported maternal health outreach. As health workers, we saw that the CSC motivated collective action. This reduced our workload and improved patient experiences. Mothers especially appreciated the changes, as the facility became more welcoming. The collaboration built through the CSC has made service delivery more effective and trusted. Male CHO, Gushegu |
|  | One key impact of the CSC was accountability. After discussions, the community took responsibility for improving services. They supported maternal health initiatives, built small structures for patients, and repaired broken equipment. This sense of ownership was new. It demonstrated that service delivery is not just for health staff, but a shared responsibility. The improvements in antenatal care and immunizations are clear outcomes of these collaborative actions following the CSC process. Female CHO, Gushegu |
|  | In Gushegu, the CSC made gaps visible, especially in maternal health and outreach. The community responded by renovating facilities and helping with staff housing. This practical support improved staff motivation and expanded our ability to deliver services. Families, particularly women, now attend services more regularly. The CSC also made our work more transparent, as the community tracks progress. The combination of accountability and resource mobilization has strengthened overall service delivery in this district. Female Health Staff, Gushegu |
|  | The CSC highlighted weaknesses such as inadequate space and lack of maternal health equipment. The community organized themselves to improve the facility. They constructed shade structures and supported water supply improvements. These contributions had a big impact on service delivery. Now more patients come willingly, and staff feel recognized. The CSC shifted health from being seen only as government’s role to a shared responsibility. This has made our work more manageable and responsive. Female Health Staff, Gushegu |
|  | One of the visible changes after CSC in Gushegu was improved maternal care. The community provided support to repair the maternity block and helped staff with housing needs. These actions increased trust, as people saw that their concerns were taken seriously. It also made our services more reliable, with improved patient comfort. Although challenges remain, the CSC helped bring the community and staff closer, which has had a long-term impact on health delivery. Female Health Staff, Gushegu |
|  | The CSC allowed us to prioritize services more effectively. With community support, we expanded maternal and child health outreach and improved hygiene at the facility. This resulted in higher attendance for antenatal and child immunizations. The process also improved accountability, because now the community expects us to report on changes. Staff motivation increased, as the facility became better equipped. The CSC has been a real turning point in strengthening services in Gushegu. Female Health Staff, Gushegu |
|  | The CSC process in Gushegu encouraged dialogue between staff and community members. The result was joint action to address gaps such as water access and facility repairs. Women especially benefitted, because antenatal care and delivery services became more consistent. This improved health-seeking behavior, as women felt more comfortable attending services. The sense of accountability created through the CSC has sustained community interest and continued improvements, which has positively influenced the quality of service delivery. Female Health Staff, Gushegu |
|  | After the CSC, we saw improvements in both infrastructure and service uptake. The community supported us by providing funds for small renovations and helping with outreach. This eased some of our workload. Patients noticed the changes and responded positively, especially in maternal and child health. The CSC helped build mutual trust between staff and the community. Service delivery became more effective because everyone felt responsible for maintaining and improving the facility’s operations. Male Health Staff, Gushegu |
|  | CSC discussions brought out the most urgent needs, such as maternity care and facility conditions. The community took steps to address them by contributing funds and labor. These changes increased patient confidence and encouraged regular service use. For staff, the improvements meant a more functional working environment. Women appreciated that antenatal care was prioritized. Overall, the CSC not only improved services but also created a stronger partnership between health workers and the community in Gushegu. Male Health Staff, Gushegu |
|  | The CSC process transformed how we engage with our community. Once gaps were identified, people mobilized resources to renovate the facility and support maternal care. These changes motivated staff and improved patient attendance. The facility is now seen as reliable, especially for antenatal and child health services. The CSC showed that collaboration and accountability can turn feedback into real improvements, making services stronger and more responsive to the needs of the people. Male Health Staff, Gushegu |
|  | After the CSC, the community built a maternity block. This came from their own mobilized resources, which directly improved maternal care. The recommendations helped us prioritize antenatal services, though not all CHPS staff were equally engaged in follow-up actions. Male CHO, Mion |
|  | The CSC showed gaps in family planning and malaria control. As staff, we received more support from the community, especially in supplying mosquito nets. However, since not every CHO was trained, implementation depended on just a few individuals. Male CHO, Mion |
|  | Service delivery improved once the CSC results were discussed openly. The community contributed funds for staff housing, which motivated us to remain in the zone. Yet, lack of full training limited consistency in applying CSC recommendations. Male CHO, Mion |
|  | The CSC can bring change, but only if there is leadership, community commitment, and follow-up support. Otherwise, it remains just a discussion tool. The maternity block construction was a turning point. It came directly from CSC findings, where community members realized women faced difficulties during delivery. The change boosted trust in the CHPS system and motivated us to expand maternal health education. Male CHO, Mion |
|  | Family planning uptake increased after the CSC process identified it as a neglected area. We organized more education sessions with women groups. Still, the challenge is sustaining these efforts since resources are not always available. Male CHO, Mion |
|  | The CSC opened dialogue between staff and community members. People understood the need to support health workers, leading them to provide water storage tanks. These contributions helped reduce service gaps, though the system still relies heavily on community goodwill. Female CHO, Mion |
|  | After the CSC, the community worked with us to improve sanitation around the facility. Building shade structures and ensuring latrines were maintained made a difference. The improvements, however, varied depending on which CHMC members were active. Female CHO, Mion |
|  | The CSC made service gaps visible. The community now pays more attention to antenatal and child health programs, supporting logistics where possible. While improvements are clear, knowledge transfer remains weak because few staff were trained directly. Female CHO, Mion |
|  | We saw visible change after the CSC recommendations. Residents offered building materials for staff accommodation, improving our presence and service delivery. The process also encouraged collective problem-solving, though sustaining it requires ongoing leadership. Male Health Staff, Mion |
|  | Malaria control improved after CSC discussions highlighted it as a priority. With community support, we organized net distribution and awareness campaigns. Yet, without consistent training for all staff, activities sometimes depend on the initiative of only a few. Male Health Staff, Mion |
|  | The CSC process created accountability. Community leaders ensured follow-up on maternal care gaps, even fundraising to improve maternity space. Still, service delivery improvements were uneven, depending on the commitment of local committees. Male Health Staff, Mion |
|  | The CSC raised expectations from the community. People now request feedback on what has changed after assessments. While we see better family planning coverage, limited external support means we still struggle to address broader gaps. Male Health Staff, Mion |
|  | Small but meaningful improvements happened after the CSC. Shade structures and water facilities reduced workload and improved patient comfort. However, the improvements depend on active community members, not always on systemic support. female Health Staff, Mion |
|  | Service delivery improved moderately through community-driven initiatives. The maternity block and water supply changes show the power of CSC. Yet, lack of comprehensive training creates bottlenecks, as not all staff know how to apply recommendations. Female Health Staff, Mion |
|  | We hear of the CSC from other districts, but in Nanton, no training or follow-up ever reached us. Without that foundation, service delivery has not improved. We continue facing the same shortages in maternal health, and community expectations remain unmet. Male CHO, Nanton |
|  | In our district, there is talk of CSC but no visible action. We still lack proper space for antenatal care and no community contributions have been mobilized. It feels like we are left behind compared to others. Male CHO, Nanton |
|  | There have been no service delivery changes linked to CSC here. We attend meetings but nothing concrete follows. Without training or resources, CSC recommendations remain abstract, and the community feels discouraged when they see no visible progress. Male CHO, Nanton |
|  | Our challenge is that while we know CSC can identify gaps, we have never used it. The health facility still struggles with basic infrastructure, and staff motivation is low because no real support comes after discussions. Female CHO, Nanton |
|  | Nothing has changed in Nanton because no CSC recommendations were ever implemented. The community asks why they should attend meetings if the issues they raise never translate into service improvements. It creates a disconnect between us and them. Female CHO, Nanton |
|  | The CSC remains only theoretical for us. We were not trained, and therefore we cannot link it to service delivery. As a result, maternal health, family planning, and malaria services continue with the same old challenges. Male Health Staff, Nanton |
|  | In this district, no CSC-based changes have been recorded. Our CHPS zones still lack staff housing and reliable water supply. Staff feel forgotten, while the community’s voice in planning is weak since feedback is not acted upon. Male Health Staff, Nanton |
|  | We expected CSC to drive improvements like in Gushegu or Yendi, but in Nanton, nothing has been adopted. People get frustrated because their concerns remain the same: lack of drugs, poor infrastructure, and limited follow-up from higher levels. Male Health Staff, Nanton |
|  | I have seen reports from other districts about CSC improving services, but for us, there is no such story. We remain in the same conditions, without additional support, which discourages both staff and community members. Male Health Staff, Nanton |
|  | The lack of CSC training and follow-up makes service delivery here stagnant. We have many gaps, from maternal care to sanitation, but there is no platform to turn these into actionable steps. It is a missed opportunity. female Health Staff, Nanton |
|  | No visible improvements came from CSC in this district. We continue improvising to manage maternal and child health. Without training or resources, the tool is irrelevant here, leaving us disconnected from progress in other districts. female Health Staff, Nanton |
|  | We hear about communities building blocks or shade structures elsewhere, but Nanton has nothing similar. The absence of CSC recommendations means no accountability mechanisms exist, and our service delivery struggles persist. Female Health Staff, Nanton |
|  | The CSC could have been useful, but since there was no training or adoption here, nothing has changed. Our staff continue facing the same workload pressures without the support or improvements reported in other areas. Female Health Staff, Nanton |
|  | The situation in Nanton shows what happens when CSC is absent. There is no improvement in infrastructure, no mobilization of resources, and no real sense of accountability. Staff feel unsupported, and the community has lost confidence. Female Health Staff, Nanton |
|  | After the community scorecard, we saw changes, more attention to family planning, antenatal care, and malaria control. The community also contributed funds to improve facilities, which motivated us as staff. But some smaller zones in the district still lack resources to match these improvements. Male CHO, Yendi |
|  | The CSC process helped us focus on malaria control and maternal care. Community members provided chairs and supported building improvements. Staff feel more valued. However, not all facilities received equal attention, so while progress is visible, gaps remain in remote areas. Female CHO, Yendi |
|  | Using CSC, the community identified shortages in antenatal services. Soon after, extra beds were provided, and families helped with minor renovations. These steps built trust. Still, full coverage was not achieved, and some CHPS compounds continue operating with limited resources. Female CHO, Yendi |
|  | The CSC encouraged dialogue between staff and community. We received more focus on immunization and child health services. The improvements gave mothers confidence to come regularly. Yet, some facilities still lack equipment for safe deliveries, showing that the impact is uneven across the district. Female CHO, Yendi |
|  | The most visible change was community contributions after CSC. They provided cement to fix cracks in the maternity block and supported outreach activities. This created strong collaboration. However, follow-up support from higher levels is still limited, which hinders sustainability of these gains. Female CHO, Yendi |
|  | After CSC discussions, mosquito nets and malaria control were prioritized. Households became more proactive, and the facility gained recognition. Still, supply chain gaps remain. Sometimes, even though the community supported, the district health system could not provide necessary logistics. Male Health Staff, Yendi |
|  | Community trust improved greatly after CSC. Mothers now see the CHPS as a reliable first point of care. They helped build a shade structure for waiting mothers. But in more distant zones, little changed, showing improvement is not yet uniform across Yendi. Male Health Staff, Yendi |
|  | With CSC, service delivery improved in areas like family planning and immunization. Communities took part by contributing small funds. This motivated us to work harder. However, there is no structured follow-up, and progress depends heavily on community initiative rather than district-level support. Male Health Staff, Yendi |
|  | The CSC highlighted poor conditions in our facility, and soon repairs were done. Community members organized contributions and fixed the roof. These small but vital changes increased staff morale. Still, without external support, more technical issues like equipment shortages remain unresolved. Male Health Staff, Yendi |
|  | The changes after CSC gave us confidence. More women came for antenatal care, and outreach coverage expanded. Yet, the improvements were not universal. Some CHPS zones in hard-to-reach areas have not benefited equally, which makes progress uneven within the district. Male Health Staff, Yendi |
|  | The CSC promoted accountability. Leaders in the community began asking about service quality, and we were able to respond with small but visible changes. This improved confidence. However, lack of drugs remains a challenge that local contributions alone cannot fix. Female Health Staff, Yendi |
|  | We saw positive change in maternal health service delivery after CSC. Women felt more encouraged to attend ANC sessions. The community supported by providing water storage. Still, broader issues like staffing gaps and inadequate delivery equipment remain unresolved at the facility level. Female Health Staff, Yendi |
|  | The CSC process gave us a platform to discuss service gaps openly. It resulted in more attention to malaria and child health. But sustaining change is difficult because there is no consistent financial support beyond what communities mobilize themselves. Female Health Staff, Yendi |
|  | Family planning services improved after CSC. The community began encouraging men to support their wives. This was a big shift. Yet, without consistent district support, the improvements may not last, as communities alone cannot provide essential supplies or staff incentives. Female Health Staff, Yendi |
|  | The CSC has transformed how the community relates to CHPS. People see it as their first choice, which was not the case before. They supported renovations and bought benches. However, not all zones received similar attention, and some continue with minimal change. Female Health Staff, Yendi |
|  | We attend meetings where CSC is discussed, but nothing happens afterward. Staff feel discouraged because there are no resources or leadership follow-up. The community raises concerns, yet no action is taken. Without improvements, trust is slowly eroding, and staff feel stuck repeating the same complaints. Female CHO, Karaga |
|  | The CSC was introduced to us, but there was no training or follow-up. We have no idea how to use it properly. When communities ask about improvements, we have no answers. This creates tension because expectations were raised but not matched with real changes. Female CHO, Karaga |
|  | Nothing has changed since the CSC process was mentioned. We lack equipment, drugs, and staff, but these issues remain. Staff feel powerless because leadership does not act. The community sees no difference, so interest in participating has started to decline. Female CHO, Karaga |
|  | We have never been trained on CSC, yet we are expected to apply it. No improvements came from the process. Communities complain about the same problems, but we cannot respond. It feels like an empty exercise without results, which reduces confidence in the CHPS compound. Female CHO, Karaga |
|  | The CSC raised expectations among community members, but no action followed. We still lack delivery kits and basic supplies. Mothers complain about long waiting times, and staff are stretched. Without leadership or resources, CSC feels meaningless, and staff are frustrated by repeating discussions without outcomes. Female CHO, Karaga |
|  | Every time we hear about CSC, it ends at the meeting level. No staff were trained, and no resources are provided. We feel disconnected from the process. Communities ask why there are no improvements, but we have no way of addressing their concerns. Male Health Staff, Karaga |
|  | We attend meetings and hear about the CSC, but nothing changes in our CHPS. There is no follow-up, no training, and no resources to act. Without training and funding, nothing moves forward. This leaves both staff and communities dissatisfied, and it undermines trust in health services. Male Health Staff, Karaga |
|  | For us, CSC has been only talk. We did not get training, and nothing has improved. Communities keep asking for water, better delivery care, and drugs, but no support comes. Staff morale is very low because there is no way to act on recommendations. Male Health Staff, Karaga |
|  | No visible change has come from CSC in Karaga. We lack staff, equipment, and housing, yet these problems remain the same. Communities feel excluded because their input leads nowhere. For staff, it feels like an exercise that raises hope but delivers nothing practical. Male Health Staff, Karaga |
|  | The problem is leadership. Without training or follow-up, CSC has not brought results. Communities expected improvements but saw nothing. This creates frustration for us as health workers because we are seen as responsible, but we have no resources or authority to implement changes. Female Health Staff, Karaga |
|  | The CSC discussions have not translated into service delivery improvements. Our facilities continue to operate with the same shortages. Staff are demoralized, and community trust is declining. Without district support, CSC cannot make any real difference here. Female Health Staff, Karaga |
|  | We have never been trained, so CSC is not functional in Karaga. The community hears about it but sees no changes. This disconnect creates tension between health workers and residents. Without proper training and resources, CSC recommendations cannot be applied. Female Health Staff, Karaga |
|  | The lack of improvements after CSC has left us frustrated. Nothing in service delivery has changed, and we continue to struggle with shortages. Staff feel unsupported, and the community has stopped expecting meaningful action from these processes. Female Health Staff, Karaga |
|  | CSC has not improved services in Karaga. We still face the same shortages of drugs and delivery kits. The process feels empty, as no one follows up on the recommendations. This leaves staff frustrated and communities disillusioned with the CHPS system. Female Health Staff, Karaga |
|  | CSC feels irrelevant here. No staff training was provided, and no service improvements have followed. Communities raise issues, but without resources, nothing changes. This constant cycle of complaints without results has weakened community engagement and reduced our motivation as staff. Female Health Staff, Karaga |
|  | After CSC, we saw small but important improvements, like better drug supply and more attention to antenatal care. The community trusted us more because they saw changes. But women and youth were not meaningfully included, and without their voices, the process does not fully address equity gaps. Male Health Staff, Sagnarigu |
|  | The CSC created momentum for maternal and child health improvements. More mothers now come for ANC because staff availability increased. However, inclusion was weak. Women and persons with disabilities were rarely engaged in discussions, and decisions remained dominated by men. This limits the equity potential of CSC here. Male Health Staff, Sagnarigu |
|  | Our compound benefited from CSC because basic equipment was provided through community contributions. It motivated staff to serve better. Yet, no training addressed how to involve women and vulnerable groups. Service delivery improved, but planning processes remain male-dominated, which reduces community ownership for certain services. Male Health Staff, Sagnarigu |
|  | The CSC outcomes were mixed. On one side, we gained community trust and better support for malaria control activities. On the other, equity concerns were ignored. Female volunteers were very few, so mothers were reluctant to discuss sensitive issues. The lack of female representation remains a problem. Male Health Staff, Sagnarigu |
|  | We can say CSC worked to improve trust and drug availability. The community now engages with CHPS more positively. Still, women and youth were not empowered in the process. Their absence means key maternal health needs or youth health issues are sometimes overlooked in planning and implementation. Male Health Staff, Sagnarigu |
|  | After CSC, our outreach services improved slightly because the community helped provide transport support. But the sessions never seriously engaged women or minority groups. Men dominated, and their priorities shaped recommendations. Without equity-sensitive participation, the process did not achieve its full purpose. Male Health Staff, Sagnarigu |
|  | The CSC made some difference, especially with better community contributions to support services. People were encouraged to come for care. But no gender lens was applied. Women’s participation in volunteer teams is still low, and that reduces women’s comfort in accessing certain services, like family planning. Male Health Staff, Sagnarigu |
|  | CSC results here were partly positive. We saw improved WASH services and greater community involvement in keeping facilities clean. But the absence of GESI training meant women and youth had little influence. The improvements are visible, but the process excluded the very groups it was meant to empower. Female Health Staff, Sagnarigu |
|  | Community participation improved after CSC. People began contributing materials for small renovations, which strengthened service delivery. However, the exclusion of persons with disabilities and women in planning was noticeable. They were more like passive clients than active contributors. This weakens inclusiveness. Female Health Staff, Sagnarigu |
|  | The CSC outcomes were clear: more attention was given to family planning, antenatal services, and drug supply. Still, women were not at the center of the process. Men dominated volunteer roles, so mothers sometimes hesitated to share concerns. That gap in representation remains a challenge. Female Health Staff, Sagnarigu |
|  | For us, CSC brought both motivation and frustration. Motivation because service delivery visibly improved, but frustration because women and young people were not invited to decision-making spaces. Their exclusion leaves gaps, particularly in adolescent health and maternal services. Improvements were partial, not comprehensive. Female Health Staff, Sagnarigu |
|  | CSC created space for community dialogue, and improvements followed in outreach activities. Yet, culturally, women could not fully participate, and youth were rarely invited. Equity issues were overlooked, and without female role models in CHMCs, service delivery cannot fully address maternal and child health. Female Health Staff, Sagnarigu |
|  | One positive change after CSC was that drug stockouts reduced because community leaders supported procurement. Service delivery became more reliable. But exclusion persisted. Youth, women, and people with disabilities were not seen as contributors. CSC gave results, but without inclusivity, the process remains incomplete. Female Health Staff, Sagnarigu |
|  | The CSC changed our approach to family planning and antenatal care. We saw higher turnout, which was encouraging. Yet, the lack of female volunteers limited how women engaged with us. They felt shy discussing private issues with men. That shows CSC outcomes are undermined without proper gender integration. Female Health Staff, Sagnarigu |
|  | The improvements from CSC cannot be denied. More attention is now given to maternal and child health, and the community is supportive. But exclusion is evident. Women, youth, and vulnerable groups are hardly engaged in planning. For CSC to be fully effective, inclusivity must go hand in hand with improvements. Female Health Staff, Sagnarigu |
| How do technical, logistical, and organizational barriers limit the effective use of DHIMS2 and RMNCAH platforms by CHPS staff? | In Gushegu, we received orientation on collecting CSC data, but not on uploading into DHIMS2. I still depend on paper forms, which are passed to the sub-district office. Sometimes these are delayed or misplaced. Without proper computer training, I cannot update the RMNCAH platform myself. When the internet connection is poor, staff give up trying, which widens reporting gaps and prevents timely updates for maternal and child health services. Male CHO, Gushegu |
|  | At my CHPS compound, only one colleague knows how to operate the computer system. When that person is away, we cannot enter anything into DHIMS2. I have tried to learn but there was no formal training or login access provided. The situation discourages us because data collection is done, but uploading is inconsistent. The slow network here makes the process even more frustrating, and our work feels incomplete without system entry. Male CHO, Gushegu |
|  | We depend heavily on the district office because we lack direct access to DHIMS2. At the facility, we compile CSC results, but since we cannot log in, the reports sit until someone from the district comes. This reduces timeliness and creates a backlog. I think the system would improve if each CHPS was given access credentials and adequate digital training, because now it feels like we are excluded from a key responsibility. Male CHO, Gushegu |
|  | I was trained to gather CSC data, but there was no follow-up on how to enter the information digitally. We prepare paper summaries and send them to the sub-district, where uploading sometimes takes weeks. The network problem in Gushegu is also common; when the system freezes, people just abandon the process. It becomes frustrating because the data is collected accurately but never reflected in DHIMS2 or RMNCAH in a timely manner. Male CHO, Gushegu |
|  | Even though I understand the importance of reporting, my challenge is the lack of equipment. In our CHPS zone, we do not have a functioning computer, so everything is written on paper. I rely on others at the district office to handle the digital part. This creates dependence and reduces accountability. If computers and reliable connectivity were provided, many CHOs like me could update RMNCAH directly without waiting unnecessarily. Female CHO, Gushegu |
|  | Our facility struggles with poor internet service. Whenever we attempt to upload CHAPs into the RMNCAH system, it is almost impossible because the page does not respond. Staff members become demotivated and prefer sending paper-based reports to the district office. This situation affects data accuracy and timeliness. We need both stronger infrastructure and more staff trained in digital entry to improve reporting. Currently, it feels like our efforts stop halfway. Female Health Staff, Gushegu |
|  | Only two people in our sub-district are familiar with DHIMS2, and all others must depend on them. When they are unavailable, no reports are uploaded, even if deadlines are near. This centralization makes the process fragile and unreliable. I personally have not received proper training on the RMNCAH platform. Giving wider access and decentralizing the skill base could reduce delays and improve the consistency of digital reporting across Gushegu. Female Health Staff, Gushegu |
|  | The main bottleneck is not just internet or training but the procedures at district level. Even when we submit our paper reports on time, uploading is delayed because of internal reviews. This leads to mismatches between when services are provided and when they appear in DHIMS2. For health planning, this creates challenges. Staff feel frustrated because it looks like we are not working, even though we are delivering care every day. Female Health Staff, Gushegu |
|  | Sometimes we are blamed for late submissions, but in truth we submit early. The issue lies in uploading at the district level. I believe the problem is partly because access credentials are restricted to a few people. Without wider training and more computers at the CHPS compounds, it will remain difficult to integrate CSC results and CHAPs into national platforms effectively, especially here in Gushegu where resources are already limited. Female Health Staff, Gushegu |
|  | In Gushegu, we use a manual process for most of our reporting. Although we hear about DHIMS2 and RMNCAH, only specific staff at the district enter the information. I often wonder why CHPS staff are not empowered with direct access. Relying on intermediaries means reports are delayed or sometimes never entered. To me, the digital platforms could help decision-making, but only if we are enabled to use them directly. Female Health Staff, Gushegu |
|  | Our challenge is compounded by lack of computers. At my health center, there is no dedicated machine for data entry. Even when trained staff are available, they cannot perform the task because of resource shortages. We use our personal phones sometimes, but connectivity is unreliable. This shows the gap between the design of the digital platforms and the realities in rural Gushegu, where electricity, devices, and internet are all unpredictable. Male Health Staff, Gushegu |
|  | I feel the DHIMS2 system is not user-friendly for staff at our level. The forms are complicated, and without regular training, it is hard to keep up. Most people prefer to hand over paper reports and disengage from the digital process. This limits accountability and motivation. For RMNCAH especially, the complexity of the platform discourages participation. Many staff agree that unless more practical training is provided, we cannot manage the digital workload. Male Health Staff, Gushegu |
|  | Sometimes, even after data is submitted to the district, it does not reflect in DHIMS2. Later, when supervisors check, they assume the facility failed to report. This creates unnecessary tension between frontline staff and managers. The real issue is the gap between reporting and uploading. For Gushegu, the way forward is to decentralize data entry, provide staff access credentials, and improve connectivity so that facilities can be accountable for their own data. Male Health Staff, Gushegu |
|  | In Mion, many of us were trained on how to collect CSC data but not on uploading into DHIMS2. I usually fill paper reports, which are sent to the sub-district. Sometimes these are entered late, and by then, the data is less useful. We need basic computer training and access to the RMNCAH platform so that we can directly update maternal and child health information without relying on district officers. Male CHO, Mion |
|  | We were trained to collect the CSC data, but not all of us know how to upload it into the system. Sometimes we just send the paper reports to the sub-district and that ends it. Only a few people here know how to operate DHIMS2. When they are away, everything stops. I once tried to learn but had no login access and no formal instruction. The community expects timely reports, but we are stuck with paper. If Mion facilities were given full access, the reporting would be better. Right now, we depend on too few staff, which creates delays and frustration across the whole CHPS system. Male CHO, Mion |
|  | Uploading CHAPs into RMNCAH is not happening consistently in Mion. The main reason is that our internet service is unreliable. Even when we try, the system freezes or fails to load. As a result, we prepare the reports but leave the digital entry to the district office. This makes our efforts less visible and weakens accountability. Giving each CHPS zone reliable connectivity and training would help solve many of these problems. Male CHO, Mion |
|  | Most of us in Mion have never received proper training on how to use RMNCAH or DHIMS2. We know the importance of the platforms, but without computers, internet, and access credentials, we cannot participate. I submit paper-based CSC summaries to the sub-district, but uploading depends entirely on others. This creates a knowledge bottleneck and means we do not get ownership of the data that we actually collect and manage every month. Male CHO, Mion |
|  | The workload of digital reporting is centralized. In Mion, only one or two staff handle DHIMS2, and the rest of us just provide paper reports. This dependence makes the whole system fragile. When these staff are busy or unavailable, no reports are uploaded. I believe spreading the skills to more health workers and providing more computers would make digital reporting more consistent, reliable, and timely across the CHPS zones here in Mion. Male CHO, Mion |
|  | For DHIMS2 in Mion, the main challenge is administrative. We prepare timely reports, but the district delays uploading them because of internal procedures. This creates gaps between what is happening in the communities and what shows in the system. The delay discourages staff. It feels like the hard work we put into service delivery and documentation does not count. More autonomy at CHPS level could reduce this bottleneck and improve timeliness. Female CHO, Mion |
|  | In our facility, the internet is so unreliable that digital reporting becomes impossible. Even when staff are trained, they cannot upload into DHIMS2 or RMNCAH because the system will not load. Many health workers become frustrated and just abandon the process. This results in an incomplete national database. The government and partners should first fix connectivity before expecting full compliance with digital platforms. Right now, reporting in Mion is inconsistent at best. Female CHO, Mion |
|  | We were told that DHIMS2 would simplify reporting, but in practice it adds stress. Most of us are not computer literate, and no refresher training is given. As a result, we continue to use manual forms. Only the district officers upload data, and this leads to delays and errors. In Mion, it feels like the promise of digital systems has not been fulfilled because frontline staff like us are not fully included. Female CHO, Mion |
|  | In Mion, health staff often lack computers. Our CHPS zone does not have a functional machine. So, even those who are trained cannot practice. We depend on handwritten reports, which are later typed by someone else at the district. This duplication wastes time and can introduce mistakes. If facilities had even one shared computer with stable electricity, reporting into RMNCAH and DHIMS2 would improve greatly, reducing errors and making data more timely. Male other health Saff, Mion |
|  | I find DHIMS2 difficult to navigate. The forms are not intuitive, and without continuous guidance, it is easy to make mistakes. For that reason, most people avoid using the system. In Mion, this results in dependence on a few digitally skilled staff. That kind of bottleneck is risky. If we want sustainable improvements, every CHO and health staff should be trained thoroughly and supported with resources to handle their own digital entries. Male other health Saff, Mion |
|  | The gap in Mion is not only technical but also structural. Health workers do not have login credentials for RMNCAH. Everything is centralized at the district office. This means that even if staff are motivated, they cannot act because access is blocked. Giving login access to facilities, coupled with training and better monitoring, could transform reporting. Otherwise, data entry remains out of our control and disconnected from the realities we manage daily. Male other health Saff, Mion |
|  | Sometimes reports from Mion reach the district on time, but they remain unentered for weeks. Supervisors later claim the facility failed to report. This undermines trust between frontline workers and managers. We need more transparency and decentralization in the system. If each CHPS had direct authority to upload, there would be fewer delays and less confusion. Right now, the system unfairly makes it appear like facilities are underperforming. Male other health Saff, Mion |
|  | The RMNCAH platform is hardly used by our facility because we have no direct access. We rely completely on the district office. This removes ownership and limits our ability to track service delivery data. Even when we collect information on maternal and child health, it remains on paper. For meaningful health planning, CHPS workers in Mion must be included more directly, otherwise the platforms will remain disconnected from daily service realities. Female other health Saff, Mion |
|  | The network issue in Mion is a serious problem. When staff attempt to log into DHIMS2, the page often fails to load or disconnects. This makes it impossible to meet deadlines. Over time, people have stopped trying. We need investment in internet infrastructure as much as in training. Without reliable connectivity, digital platforms will remain underutilized, no matter how much staff are motivated or how many paper reports we generate. Female other health Saff, Mion |
|  | In Nanton, we collect the reports but nothing changes because we cannot upload into DHIMS2 ourselves. Everything is left to the district, and often there is no feedback. It feels discouraging because our work ends at writing, not reporting. We were not trained on CSC reporting or RMNCAH entry, so most of us are simply sidelined. The system gives the impression of modernity, but for frontline workers here, it has brought little improvement. Male CHO, Nanton |
|  | We face frustration every month. I prepare paper summaries, but after submission, I never know whether they are entered. No CSC improvements have been seen, and reporting is not transparent. Without training and access, our role ends at collecting data. For many of us, it feels like wasted effort. The CHPS compound should have digital authority, but instead, we are only spectators in a process we are supposed to lead. Male CHO, Nanton |
|  | Most staff in Nanton are completely excluded from digital platforms. DHIMS2 and RMNCAH are managed from the district office, and we do not even have login details. That makes us dependent and disempowered. Even when CSC highlighted service gaps, nothing was done because results were not linked to these platforms. It creates frustration for health workers and communities who expect improvements that never come due to poor data translation into practice. Male CHO, Nanton |
|  | In Nanton, many health staff have not even seen the DHIMS2 interface. We hear about it during meetings, but in practice, we are not involved. This has made CSC reporting ineffective because no data reaches the higher levels directly from us. Without training, internet, or computers, CHPS zones cannot contribute. The lack of ownership discourages participation, and after a while, staff become passive because they know their efforts won’t be recognized. Female CHO, Nanton |
|  | The challenge is both technical and systemic. In Nanton, even when we send reports, they are delayed or not uploaded. The district does not share updates, so we do not know what has been entered. This lack of communication undermines trust in the system. Without access to platforms, our CSC findings remain invisible. The whole point of community scorecards is lost if results cannot influence planning or be integrated into national systems effectively. Female CHO, Nanton |
|  | I feel the system in Nanton is not working. We depend on paper-based reporting, but there is no guarantee it reaches the database. Staff do not have computers or internet. We were never trained on DHIMS2 or RMNCAH, so there is no confidence. This creates disillusionment. Communities are told their inputs will inform decisions, but without a functional digital link, the CSC process becomes only talk, with no follow-through in practice. Male Health Staff, Nanton |
|  | The CSC raised issues of maternal health services, but because reports were not uploaded, no changes happened. As staff, it is demoralizing. We send paper reports, and that is the end of it. If DHIMS2 was accessible at CHPS level, improvements might follow. For now, Nanton remains disconnected. The community starts to lose trust, seeing that their concerns are documented but never acted upon. This weakens participation and makes our role more difficult. Male Health Staff, Nanton |
|  | In Nanton, the lack of training is clear. Most of us cannot use a computer for reporting. Even when reports are ready, we must rely on district staff. This dependence creates delays, and often reports are not entered at all. The system looks modern, but at our level, it is ineffective. Without CSC-driven improvements, it feels like data collection is just for show, and communities notice that nothing tangible comes out of it. Male Health Staff, Nanton |
|  | Digital reporting has created more distance instead of helping. We in Nanton do not have access to RMNCAH or DHIMS2. Our facilities lack the equipment and credentials. Even when community members provide feedback through CSC, it is lost in the process. We are frustrated because improvements depend on data visibility, yet the tools to make that happen are beyond our reach. The system must include CHPS workers, or it will remain ineffective. Male Health Staff, Nanton |
|  | The digital platforms in Nanton are controlled from the top, so local health staff are powerless. Our role is reduced to writing reports that are hardly used. CSC findings pointed to gaps, but no changes came because they were never uploaded. The lack of access also means we cannot check the accuracy of entries. This makes staff lose confidence, and eventually, we just do the bare minimum since results are never acknowledged. Female Health Staff, Nanton |
|  | Nanton has become a case of exclusion. We prepare reports diligently, but without access to digital systems, the work is incomplete. Even when we highlight urgent maternal or child health concerns, they are lost in the pile. The frustration is not only among staff but also among community members, who ask why nothing improves. Unless reporting is decentralized, CHPS zones here will always remain invisible in the national health information system. Female Health Staff, Nanton |
|  | One of the biggest frustrations in Nanton is the absence of feedback. We send data, but nothing comes back. It makes staff feel disconnected from decision-making. The digital platforms are supposed to improve accountability, but without our participation, they instead create barriers. CSC was meant to bring communities and health staff closer to planning, but without integration into DHIMS2 and RMNCAH, the whole process has little value at the facility level. Female Health Staff, Nanton |
|  | In our CHPS, we don’t even try to use digital systems anymore. We know we lack the training and access, so we focus on paper-based reporting. The district takes responsibility, but delays and errors mean that our inputs don’t appear on time. It is frustrating to work hard on CSC data collection when we know it won’t be translated into improvements. Communities now hesitate to participate because they feel their voices don’t matter. Female Health Staff, Nanton |
|  | The system in Nanton is broken at the facility level. We do not have computers, training, or access rights. As a result, DHIMS2 and RMNCAH remain abstract platforms we cannot engage with. This leaves CSC results unused, even though they identified important gaps. Staff morale suffers because the reporting burden feels meaningless. To make progress, access and responsibility must be shifted closer to CHPS facilities rather than left only at the district office. Female Health Staff, Nanton |
|  | After the CSC, we saw real improvements. Family planning services were prioritized, and antenatal care visits increased. The community supported us by repairing the facility roof and contributing for benches. This motivated staff greatly. Still, we lack enough midwives, so some maternal health challenges remain despite the progress. Male CHO, Yendi |
|  | The CSC made our gaps clear, and leadership responded. Malaria control activities improved, with more bed nets distributed and awareness sessions held. Community members also helped construct a small waiting area. However, some recommendations like staffing additional nurses have not been achieved, so improvements remain partial. Female CHO, Yendi |
|  | In Yendi, CSC changed the way people see CHPS. The community trust increased because they saw action. More mothers now come for antenatal care and delivery. But challenges persist, particularly in drug shortages. Community contributions addressed infrastructure gaps, but supply issues remain unresolved, limiting the full impact of the process. Female CHO, Yendi |
|  | The scorecard encouraged transparency. We had open discussions with the community, and they agreed to support water provision for the CHPS compound. This improved hygiene for staff and patients. However, equipment for diagnostics is still missing, meaning some referrals continue unnecessarily. The improvements are visible but incomplete. Female CHO, Yendi |
|  | With the CSC, we became more accountable to the community. People noticed that their feedback mattered, as family planning services and ANC clinics were strengthened. Yet, we still struggle with shortage of midwives. Women sometimes wait long for delivery care. The progress is real but not consistent across all service areas. Female CHO, Yendi |
|  | CSC opened space for communities to directly shape services. After discussions, funds were raised for a maternity block extension. This was a major boost for maternal health delivery. However, gaps still exist in logistics and supply chains. It shows that while CSC is powerful, results depend on resources to implement changes fully. Male Health Staff, Yendi |
|  | In Yendi, we noticed the biggest change in malaria services. CSC highlighted it, and now community members are more engaged in net usage and outreach. ANC attendance also improved. But there are still equity gaps young people and vulnerable groups were not fully considered in the improvements, which limits inclusiveness. Male Health Staff, Yendi |
|  | The CSC process motivated the community. They supported staff housing, making our work easier. This has reduced absenteeism. Family planning uptake increased as well. Still, some recommendations like adding laboratory services were not implemented due to resource constraints. It shows progress but also the limits of what local action alone can achieve. Male Health Staff, Yendi |
|  | CSC brought accountability. We used to complain about lack of drugs, but now the district makes more effort to ensure supplies. Community members also monitor stockouts. However, certain essential items still run out. Improvements are clear in maternal health, but for chronic conditions, less attention was given after the scorecard. Male Health Staff, Yendi |
|  | Community involvement increased after CSC. They even mobilized money to repair the facility fence, improving security. This built trust with staff. But we still lack digital reporting capacity, so feedback is not always integrated into DHIMS2. Without that link, some improvements stop at the community level and don’t reach policy discussions. Male Health Staff, Yendi |
|  | The scorecard process made the community see us differently. They became partners, not just patients. Women’s attendance at ANC improved, and family planning conversations are now more open. Still, youth needs were less prioritized, and their concerns about recreational services and sexual health were not translated into actions. Female Health Staff, Yendi |
|  | After the CSC, we saw visible infrastructure upgrades. The community helped to build shade structures for waiting mothers. It reduced stress for both patients and staff. However, despite these successes, staffing shortages remain. The CSC identified it clearly, but without district-level recruitment, that gap cannot be filled through local initiatives. Female Health Staff, Yendi |
|  | The biggest achievement from CSC was that it motivated communities to co-own the CHPS compound. They cleaned the surroundings, supported outreach, and contributed resources. This improved trust. But we still face challenges with specialized services like nutrition and laboratory testing, which the CSC identified but remain unaddressed due to limited district support. Female Health Staff, Yendi |
|  | Yendi has seen strong CSC benefits. Malaria services, ANC, and delivery care all improved. The community contributed resources, showing genuine ownership. Still, the changes are not uniform across all CHPS zones. Some facilities have seen major improvements, others little. Sustainability remains uncertain without consistent district leadership and follow-up support. Female Health Staff, Yendi |
|  | CSC changed the relationship between staff and community. Before, they complained about services without understanding constraints. Now, they work with us to solve problems. We got help with water, sanitation, and family planning outreach. But some issues like staffing and drug shortages remain unresolved. So, while positive, the improvements are still incomplete. Female Health Staff, Yendi |
|  | Some of the reports are submitted on time, but because of internal procedures, they are not uploaded into DHIMS2 immediately. That creates gaps in the system. Male CHO, Karaga |
|  | The CSC was conducted, but we saw no follow-up. Family planning and ANC services remain under-resourced, with no new support or staff deployment. Women still complain of long waits. Without leadership acting on the scorecard, the exercise became meaningless. Communities now view us as powerless to make improvements. Female CHO, Karaga |
|  | Unlike other districts, Karaga saw no visible change after CSC. The same shortages continue: no essential drugs, insufficient staff, and poor infrastructure. Community members ask why they should participate if nothing is implemented. This discourages both providers and users, eroding the trust that CSC was supposed to build. Female CHO, Karaga |
|  | We presented clear challenges during CSC, such as lack of midwives and poor facility structures. But there was no district-level response. The same problems remain. This made community members angry with health staff, as if we ignored their needs. In truth, we had no power to implement the recommendations. Female CHO, Karaga |
|  | CSC here ended up raising expectations without action. People expected better ANC and delivery services, but nothing improved. We still operate with very few staff, no reliable transport, and limited supplies. Instead of motivating communities, it created disappointment. Participation in health meetings is now declining steadily. Female CHO, Karaga |
|  | We depend on one or two staff who know how to use the computer. If that person is not around, nothing goes into DHIMS2. The process left us worse off because it exposed gaps but offered no solutions. Women still travel far for delivery care. Community members feel excluded because their input was never acted on. Staff morale dropped, as the same issues, drugs, infrastructure, and staff shortages remain exactly as before the CSC. Male Health Staff, Karaga |
|  | In Karaga, the CSC failed to trigger change. Even basic recommendations like repairing the facility roof were ignored. People accuse health staff of making empty promises. This has created tension between staff and community. Without action from leadership, CSC became an exercise in frustration rather than a solution. Male Health Staff, Karaga |
|  | The CSC did not bring improvements. Instead, it exposed needs that remain unresolved. For example, malaria services remain weak, and drug stockouts are frequent. Community contributions were never mobilized, unlike in other districts. Now people avoid meetings, saying it is a waste of time. The trust gap widened. Male Health Staff, Karaga |
|  | After CSC, expectations were high, but no action was taken. The district health office never followed up. This disappointed the community, and now they blame us. Some refuse to engage with CHPS staff. Instead of improving accountability, the scorecard worsened relationships. It feels like the process failed here. Male Health Staff, Karaga |
|  | In Karaga, no recommendation was implemented. We still lack clean water at the facility, and sanitation is poor. Community members stopped volunteering. They say CSC was just talk without results. For staff, this reduces motivation. It has created a sense of helplessness, since we have no authority to act. Female Health Staff, Karaga |
|  | CSC meetings highlighted service gaps, but nothing happened afterward. The community is frustrated. Some people even accuse us of hiding resources. Family planning uptake declined because trust in health services has weakened. Unlike other districts, here CSC did not strengthen accountability it deepened feelings of neglect and exclusion. Female Health Staff, Karaga |
|  | The absence of follow-up made the CSC irrelevant in Karaga. Infrastructure problems persist, with no improvement in maternal or child health services. Community members openly criticize the process. Instead of building collaboration, it created conflict. Now, health committees find it difficult to mobilize people for meetings or outreach. Female Health Staff, Karaga |
|  | We tried to engage the community after CSC, but without district support, nothing improved. The same shortages exist: no staff, no equipment, poor environment. People now view CHPS compounds as abandoned structures. Staff morale is very low. Unlike other places, the CSC produced no tangible results here. Female Health Staff, Karaga |
|  | The scorecard exercise in Karaga highlighted all the problems, but no solutions were implemented. This has weakened trust. People expected at least minor changes, but none came. Staff are blamed unfairly for inaction. It feels like CSC here was only symbolic, without the real accountability or responsiveness it promised. Female Health Staff, Karaga |
|  | The platform is controlled from the district level. At the CHPS compound, we don’t have the login details, so we cannot do the entry ourselves. Female Health Staff, Karaga |
|  | CSC brought visible change here. Family planning uptake improved, and the facility received support for minor renovations. Communities also contributed to buying chairs and fixing water points. However, the process did not address equity. Women with disabilities and marginalized households remain excluded because GESI principles were not part of the training. Male Health Staff, Sagnarigu |
|  | After CSC, attention to ANC and malaria services increased. Community members provided small funds to repair broken doors and improve sanitation. This motivated staff and improved patient attendance. Still, the lack of gender and equity focus means some vulnerable groups remain underserved, showing the limitations of improvements achieved. Male Health Staff, Sagnarigu |
|  | In Sagnarigu, CSC led to new initiatives like stronger maternal health outreach and drug supply monitoring. These changes helped service delivery. But the exclusion of equity-sensitive strategies reduced impact for women with disabilities and the very poor. CSC worked, but without GESI it only partially solved the real problems. Male Health Staff, Sagnarigu |
|  | CSC recommendations helped fix small infrastructure gaps. Staff housing improved slightly with community contributions, and patients appreciated better seating at facilities. Maternal and child health care received stronger support. However, because GESI training was not provided, marginalized voices were left out, meaning changes didn’t fully reach everyone equally. Male Health Staff, Sagnarigu |
|  | CSC boosted community ownership. They helped improve WASH around the facility and supported maternal care outreach. Service utilization improved, especially ANC. But women from the poorest households still face barriers. This gap exists because GESI-responsive training was missing, so improvements, while visible, did not fully reduce inequalities in care. Male Health Staff, Sagnarigu |
|  | CSC produced concrete outcomes in Sagnarigu, unlike in some districts. For example, malaria testing and ANC services improved. The community even renovated a waiting shed. Yet, the absence of GESI meant vulnerable women were left out of decision-making. The process improved services but failed to transform equity and inclusion. Male Health Staff, Sagnarigu |
|  | The CSC recommendations resulted in better supervision and increased family planning awareness. Community leaders became more involved, and resources were mobilized locally. Still, without GESI training, disadvantaged groups were ignored. People living with disabilities received no additional consideration, so while service delivery improved overall, inclusiveness remained weak. Male Health Staff, Sagnarigu |
|  | CSC drove some positive changes. Patients reported better ANC and family planning counseling. The community also supported facility maintenance. However, these improvements mainly benefited the majority. The poor and women with special needs remain excluded because equity issues were not addressed. CSC worked, but incompletely, in the absence of GESI. Female Health Staff, Sagnarigu |
|  | In Sagnarigu, we saw improvements such as timely malaria treatment, community renovations, and stronger maternal care. Yet, the changes were uneven. The most vulnerable poor, disabled, or socially excluded still face barriers. CSC improved services generally but missed addressing inclusion because GESI-responsive training was not conducted here. Female Health Staff, Sagnarigu |
|  | The network is often slow here too. Even when we try to enter the CHAPs into RMNCAH, the system does not respond, so people give up and wait for the district office. Female Health Staff, Sagnarigu |
|  | After CSC, service delivery in family planning and ANC became stronger. Malaria testing and treatment were prioritized, and the community took part in facility upgrades. But vulnerable groups felt excluded from decisions. Without GESI training, the improvements remain partial and do not fully meet equity and gender-sensitive standards. Female Health Staff, Sagnarigu |
|  | Unlike in Karaga or Nanton, here CSC had an impact. Communities funded infrastructure repair and supported health staff. ANC coverage grew. Still, the absence of GESI meant widows, disabled women, and poor families were left behind. CSC improved services broadly but did not make them inclusive for all. Female Health Staff, Sagnarigu |
|  | CSC made health staff more responsive, and communities collaborated to strengthen maternal and child health services. However, lack of GESI training reduced inclusiveness. Many marginalized groups were left out of both planning and benefit-sharing. The progress achieved is fragile and may not last without deeper equity-sensitive strategies. Female Health Staff, Sagnarigu |
|  | In Sagnarigu, CSC boosted collaboration, and service delivery improved. The community even helped renovate a maternity block. Yet, gender and equity considerations were missing. Staff recognize this limitation. The progress was real but not holistic—women from disadvantaged backgrounds still face barriers to accessing services and participating in decisions. Female Health Staff, Sagnarigu |
|  | The CSC recommendations produced positive changes here, including better facility conditions and stronger maternal health services. But the absence of GESI training meant vulnerable populations were excluded. This created a two-tier effect: overall improvements for many, but persistent inequities for the marginalized. CSC achieved much but fell short on inclusion. Female Health Staff, Sagnarigu |
